# Estimation of foot-and-mouth disease virus sero-prevalence rates using novel computational approach for the susceptible bovine population in India during the period 2008–2021

**DOI:** 10.1038/s41598-023-48459-w

**Published:** 2023-12-19

**Authors:** Samarendra Das, Soumen Pal, Sagar Sangam Rautaray, Jajati K. Mohapatra, Saravanan Subramaniam, Manoranjan Rout, Shesh N. Rai, Rabindra Prasad Singh

**Affiliations:** 1 ICAR-National Institute on Foot and Mouth Disease, International Centre for Foot and Mouth Disease, Arugul, Bhubaneswar, Odisha 752050 India; 2 Division of Computer Application, ICAR-Indian Agricultural Statistics Research Institute, New Delhi, 110012 India; 3 College of Medicine, University of Cincinnati, Cincinnati, OH 45267 USA

**Keywords:** Computational science, Computer science, Software, Statistics, Infectious diseases, Viral infection

## Abstract

Foot-and-mouth disease (FMD) is a severe contagious viral disease of cloven-hoofed animals. In India, a vaccination-based official FMD control programme was started, which got expanded progressively to cover entire country in 2019. The serological tests are used to determine non-structural protein based sero-prevalence rates for properly implementing and assessing the control programme. Since 2008, reporting of the FMD sero-surveillance was limited to the serum sample-based serological test results without going for population-level estimation due to lack of proper statistical methodology. Thus, we present a computational approach for estimating the sero-prevalence rates at the state and national levels. Based on the reported approach, a web-application (https://nifmd-bbf.icar.gov.in/FMDSeroSurv) and an R software package (https://github.com/sam-dfmd/FMDSeroSurv) have been developed. The presented computational techniques are applied to the FMD sero-surveillance data during 2008–2021 to get the status of virus circulation in India under a strict vaccination policy. Furthermore, through various structural equation models, we attempt to establish a link between India’s estimated sero-prevalence rate and field FMD outbreaks. Our results indicate that the current sero-prevalence rates are significantly associated with previous field outbreaks up to 2 years. Besides, we observe downward trends in sero-prevalence and outbreaks over the years, specifically after 2013, which indicate the effectiveness of various measures implemented under the FMD control programme. The findings of the study may help researchers and policymakers to track virus infection and identification of potential disease-free zones through vaccination.

## Introduction

Foot-and-mouth disease virus (FMDV) is the etiological agent responsible for foot-and-mouth disease (FMD) in cloven-hoofed livestock^1^. In livestock, the FMD is characterized by acute fever with formation of vesicles in the oral cavity and feet^2,3^. The aftereffects of this disease include loss of appetite and body weight, decreased milk yield, decreased draft power, abortions, etc.^4,5^ leading to loss of livestock productivity and restrictions on trade of animals and products^6–8^. The estimated economic loss due to the FMD in its endemic regions, including Asia, South America, and Africa is ~ 6.5–21 billion USD per annum^9^. Due to the highly contagious nature, rapid spread of the infection, trans-boundary movement of animals, global trade, etc., there is a high likelihood of the spread of the virus to other parts of world, including FMD-free zones (e.g., Europe, North America, etc.)^10^.

With a bovine population of 300 million, India is the largest producer and consumer of dairy products and contributes ∼70% to total livestock income^11,12^. FMD outbreaks in India are caused by three serotypes, namely O, A, and Asia1^13^ causing an estimated economic loss up to 200 billion INR per annum^5,8,14^. For this purpose, Government of India has started mass vaccination of its bovine population with inactivated vaccines through the FMD control programme (FMDCP) and the Assistance to States to Control Animal Disease programme (ASCAD)^15^. With the implementation of the National Animal Disease Control Programme (NADCP) in 2019 (renamed as Livestock Health and Disease Control Programme (LHDCP))^16^, the whole country is now covered under the vaccination programme.

The success of disease control programme not only depends on vaccination with good quality vaccines but also on effective sero-surveillance activities^17,18^. In India, FMD sero-surveillance is being conducted regularly at the national level to assess the effectiveness of the FMDCP using the World Organisation for Animal Health (WOAH) approved indigenous serological test, i.e., non-structural protein ELISA (NSP-ELISA)^19,20^. This test is based on the principle “Differentiating Infected from Vaccinated Animals (DIVA)”, which monitors the recent past infection and virus circulation status in the susceptible vaccinated and non-vaccinated animals as well (i.e., sero-prevalence)^19,20^. Furthermore, the evidence of NSP-antibodies (NSP-Ab) is accepted as a differential marker for FMDV infection in a vaccinated population since that is expected to be induced only in case of virus infection^21,22^. This is not in case of vaccination with an inactivated vaccine which ideally shall contain only structural protein^22,23^. The DIVA approach informs about the previous or current infection and is helpful at the herd/farm level in isolating and screening the infected animals from the non-infected ones to prevent the spread of outbreaks^23–25^. This is especially true because of the persistence nature of the FMDV infections.

India has been following a two-stage sampling design since 2008 for FMD sero-surveillance^26,27^. The statistical interventions in terms of estimation methodology and software tool are still lacking to provide population-level FMDV circulation statistic. For instance, a multi-stage sampling based protocol was developed in India to estimate the population prevalence of COVID-19 infection at the national-level^28^. However in FMD, the researchers limit their interpretations to the results obtained from serum samples without going for population-level sero-prevalence rate estimation^29–31^. Many studies have found that the FMD sero-surveillance is an important step in determining the success of a vaccination programme or declaring a region as FMD-free^4,32^. For this purpose, population level sero-surveillance parameters need to be estimated from the sample data to effectively support the control programme. This necessitates advanced statistical methods and tools for estimating the sero-prevalence rate at population-level using test results from NSP-ELISA. However, attempts have been made to estimate the FMDV sero-prevalence through cross sectional sample-based sero-surveillance in cattle^29,33,34^ and small ruminants^29^ in Ethiopia, cattle and wild life in Nigeria^35^, wild life and buffalo population in eastern Africa^36^, small ruminants in Kenya^2^, etc. In all these studies, the sero-prevalence results were limited to sample proportions (e.g., total sample: 384; positive sample: 76; sero-prevalence rate: 19.79%)^29,33–36^ without going for country-level estimate of FMDV sero-prevalence rate.

Usually, sero-prevalence rates are computed from sample data without going for population-level estimation though a random sampling design based sero-surveillance has been followed in India since 2008. Therefore, in this study we present a novel statistical method for estimating the state and national-level sero-prevalence rates using the sera sample results from the NSP-ELISA test. We apply this methodology to analyse FMD sero-surveillance data of India during the year 2008–2021. Based on the developed methodology, two statistical tools (online web-application and offline R software package) have been developed and freely available for use. Furthermore, we establish the relationship between the estimates of sero-prevalence rates with current and previous field FMD outbreaks in India through various structural equation models. Besides, we observe downward trends in sero-prevalence and outbreaks over the years, which indicate the effectiveness of various FMD control measures.

## Material and methods

### Study design and data source

There are 28 states and 8 union territories (UT) in India, and each state is further subdivided into administrative units known as districts. In India, FMD control and reporting mostly rely on passive surveillance conducted by different authorities, including veterinarians and veterinary paraprofessionals (~ 65,000 at different administrative levels)^16^. Further, FMD outbreaks at village level are reported to district offices, and then a monthly report is submitted by the state to the Department of Animal Husbandry and Dairying (DAHD), Government of India. The clinical diagnosis and confirmation of the outbreaks are done through a network of 31 FMD regional and collaborating centres distributed across the country and supervised by the national FMD reference laboratory of ICAR-National Institute on Foot and Mouth Disease (ICAR-NIFMD), Bhubaneswar. These centres mostly involve themselves in activities including sample collection, testing and diagnosis, and conducting awareness campaign.

**Figure 1:**
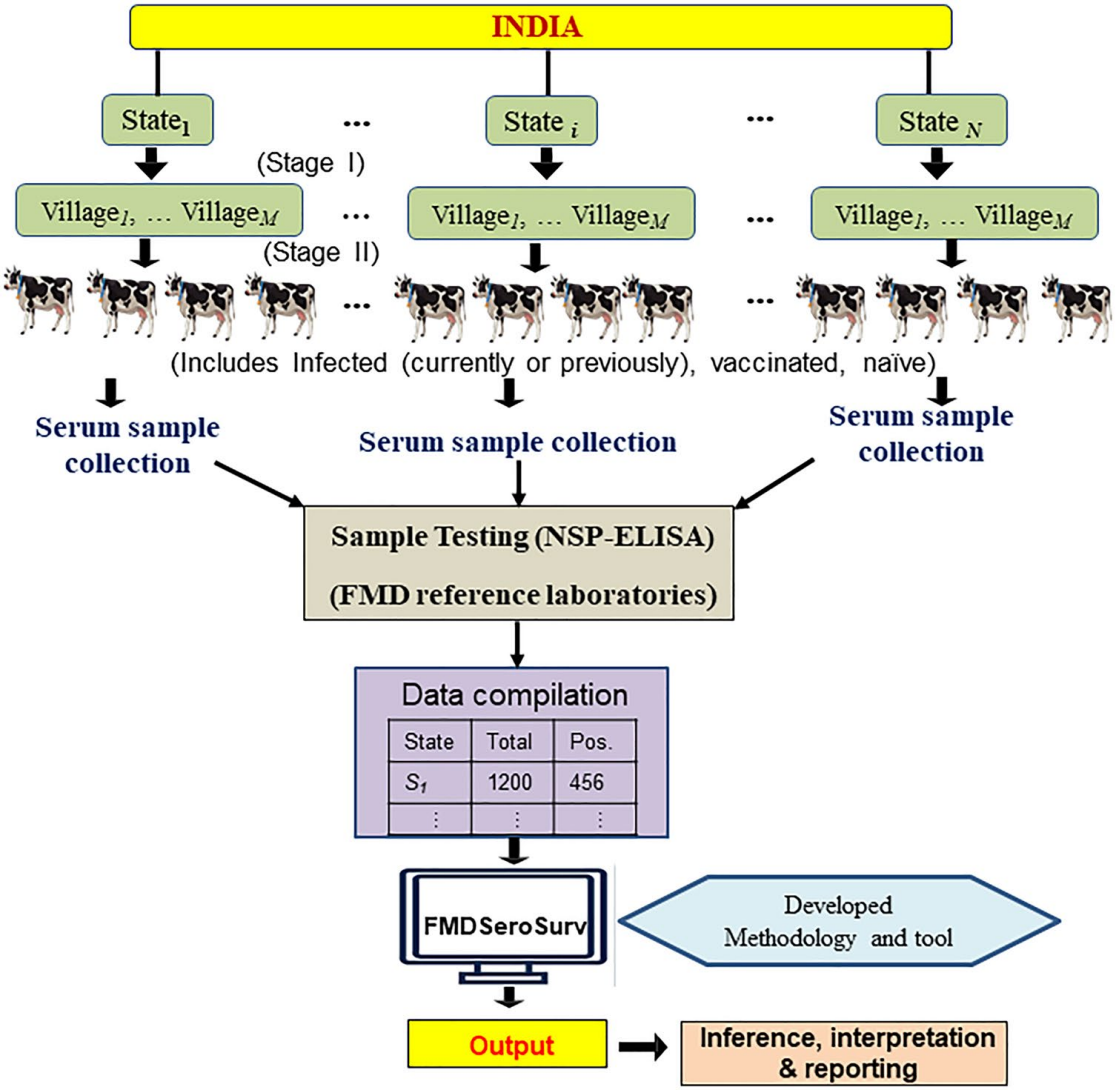
Schematic diagram of steps undertaken in this study. A two-stage sampling design was followed for national-level surveillance of FMDV in India. At stage I villages are randomly selected from the considered states and in stage II animals are selected randomly from the selected villages (at stage I) for serological test through NSP-ELISA. After obtaining the sample-level DIVA test results data through NSP-ELISA, then such data was given input to the developed method and tool to compute various state and national-level measures for FMDV sero-surveillance.

The primary method of controlling FMD in India is vaccination with inactivated trivalent vaccines^4,5,16,37^. To differentiate the infected from the vaccinated animals, India has adopted an in-house validated 3AB3-NSP-ELISA test^23^ for country-wide sero-surveillance since 2008. The diagnostic sensitivity of the test was estimated as 96%, while the diagnostic specificity varied between the naïve and vaccinated as 99.1% and 96.4%, respectively^23^. For FMD surveillance, bovine serum samples (from animals aged 6–18 months) are randomly collected from the various villages of the states. The outlines of various steps considered in this study are shown in Fig. 1. The number of states considered in the year-wise FMD sero-surveillance activity is shown in Supplementary Fig. S1. So far, a consistent strategy has been adopted for FMD sero-surveillance in India. The distribution of year-wise serum samples tested in India is shown in Supplementary Fig. S2. During 2008–2021, 0.65 million samples were tested for NSP-Ab responses, which is an indicator for FMD virus exposure regardless of the vaccination (Fig. S3).

Data on NSP-Ab prevalence for the selected random serum samples from various states were obtained from the institutional data repository of ICAR-NIFMD (http://www.pdfmd.ernet.in accessed on August 10, 2022), which is the national institute for FMD. We obtained the state-wise susceptible bovine population data on the animal census from the DAHD website (http://www.dahd.nic.in accessed on August 25, 2022). Such census studies are usually conducted in every 5 years to get the animal population of India. The distributions of the state-wise bovine population are shown for 2007, 2012, and 2019 censuses in Supplementary Figs. S3–S6 and further adjusted for age-based susceptible animal population. To study the relation between the estimated sero-prevalence rate and field outbreaks, we used the reported FMD outbreak data. The exact number of infected animals was not available for a given outbreak and was only reported at the state-level through passive surveillance. The state-level FMD outbreak data were retrieved from the ICAR-NIFMD’s data repository (2008–2021). Here, an outbreak of FMD refers to an incident where at least two animals experienced FMD illness, which are linked to spatio-temporal distribution of the disease.

### Developed methodology

#### Notation

${y}_{ij}$ is the NSP-ELISA testing result for *jth* ($j=1, 2,\dots , {m}_{i}$) (random) serum sample collected from *ith* ($i=1, 2,\dots , n$) state; ${m}_{i}$ is the number of sera samples collected from *ith* state (sample size); ${M}_{i}$: population size (i.e., total number of animals) of *ith* state; $n$: number of states considered in sero-surveillance; $N$: total number of states.

The NSP-ELISA testing results for serum sample can be defined in Eq. (1).

1 $${y}_{ij}=\left\{\begin{array}{ll}1 & \quad if \; {PP}_{ij}\ge 0.4\\ 0 & \quad if \; {PP}_{ij}<0.4\end{array}\right.$$

where, ${PP}_{ij}$ is the percent of positive value for *jth* sample collected from the *ith* state. The details of computation of PP can be found in literature^20,23,30^ and expressed in Eq. (2). In other words, ${y}_{ij}=1$ indicates that *jth* serum sample collected from *ith* state is found to be positive for FMDV infection using the NSP-ELISA test.

2 $${PP}_{ij}=\frac{Test \; serum \;sample \;mean \; OD \; value}{Positive \; control \; serum \; sample \; mean\; OD \;value}$$

It is to note that researchers always compute sample-based sero-prevalence rate without going for population-level (e.g., state) estimation^6,19,29,30^. Thus, we proposed an estimator (${p}_{i}$) for estimating sero-prevalence for *ith* state, shown in Eq. (3).

3 $${p}_{i}=\overline{{y }_{i}}=\frac{\sum_{j=1}^{{m}_{i}}{y}_{ij}}{{m}_{i}}$$

The sample variance (${s}_{i}^{2}$) for sero-prevalence rate can be expressed in Eq. (4).

4 $${s}_{i}^{2}=\frac{{m}_{i}}{{m}_{i}-1}{p}_{i}{q}_{i}$$

where, ${q}_{i}=1-{p}_{i}$ (i.e., rate of sero-negative in samples collected from *ith* state).

The ${p}_{i}$ (Eq. 3) and ${s}_{i}^{2}$ (Eq. 4) are the estimators for estimating the state-level sero-prevalence rate (${P}_{i}$) and variance (${S}_{i}^{2}$) parameter. These parameters are unknown and usually estimated from the sample data. Here, the estimators, ${p}_{i}$ and ${s}_{i}^{2}$, are unbiased for population parameters (${P}_{i}\mathrm{ and }{S}_{i}^{2}$), as shown in Eq. (5). The detailed proof of this is given in Supplementary Document S1.

5 $${E(p}_{i})={P}_{i}=\frac{\sum_{j=1}^{{M}_{i}}{y}_{ij}}{{M}_{i}}$$

6 $$E\left({s}_{i}^{2}\right)={S}_{i}^{2}=\frac{{M}_{i}}{{M}_{i}-1}{P}_{i}{Q}_{i}$$

Now, variance of the estimator ${p}_{i}$ can be expressed in Eq. (7).

7 $$Var\left({p}_{i}\right)=\frac{{M}_{i}-{m}_{i}}{{M}_{i}{m}_{i}}{S}_{i}^{2}=\frac{{M}_{i}-{m}_{i}}{{m}_{i}({M}_{i}-1)}{P}_{i}{Q}_{i}$$

In Eq. (7), the values of ${P}_{i} \; {\text{and}} \;{Q}_{i}$ are unknown, thus estimated from the sample values. Now, the estimated value of variance of the estimator (Eq. 7) becomes:

8 $$\widehat{Var}\left({p}_{i}\right)=\frac{{M}_{i}-{m}_{i}}{{M}_{i}{m}_{i}}{s}_{i}^{2}=\frac{{M}_{i}-{m}_{i}}{{M}_{i}({m}_{i}-1)}{p}_{i}{q}_{i}$$

The estimated values of the number of animals having an exposure/history of FMDV infection can be computed as:

9 $$\widehat{{Y}_{i}}={M}_{i}{p}_{i}$$

The Standard Error (SE), Margin of Error (ME), and 95% Confidence Interval (CI) of the estimator, ${p}_{i},$ can be given in Eqs. (10–13).

10 $$SE\left({p}_{i}\right)=\sqrt{\widehat{Var}\left({p}_{i}\right)}$$

11 $$ME\left({p}_{i}\right)={Z}_{0.05} SE\left({p}_{i}\right)$$

12 $$CV\left({p}_{i}\right)=\frac{SE\left({p}_{i}\right)}{{p}_{i}}\times 100$$

13 $$CI\left({p}_{i}\right)={p}_{i}\mp {Z}_{0.05} SE\left({p}_{i}\right)$$

In the FMD sero-surveillance, the villages are selected randomly from states and then NSP-antibody prevalence was observed through 3AB3-NSP-ELISA test on the selected samples from the animals (randomly selected from the selected villages). In other words, in two-stage sampling design, the primary sampling units are villages and the secondary sampling units are the animals. Hence, the adopted two-stage sampling design provides state-level sero-prevalence rate indicating the history of FMDV circulation and these estimates can be used to develop an estimator for estimating the national-level sero-prevalence rate for assessing the exposure of bovine population to FMDV. The method is briefly described below.

The estimators for sero-prevalence rate and total number of animals having some history of FMDV infection at the national level ($\tau$) is given in Eqs. (14 and 15).

14 $$\widehat{\tau }=\frac{N}{n}\sum_{i=1}^{n}\widehat{{Y}_{i}}$$

15 $$\widehat{P}=\frac{N}{nM}\sum_{i=1}^{n}\widehat{{Y}_{i}}=\frac{\widehat{\tau }}{M}$$

where, *M* is total susceptible bovine population in India across all the states, $M=\sum_{i=1}^{N}{M}_{i}$. The variance of the estimators $\widehat{\tau }$ and $\widehat{P}$ can be derived as:

16 $$Var\left(\widehat{\tau }\right)=\frac{N(N-n)}{n}{s}_{u}^{2}+\frac{N}{n}\sum_{i=1}^{n}\frac{{M}_{i}({M}_{i}-{m}_{i})}{{m}_{i}}{s}_{i}^{2}$$

where, the expressions for ${s}_{i}^{2}$ and ${s}_{u}^{2}$ are given in Eqs. (4 and 17) respectively.

17 $${s}_{u}^{2}=\frac{1}{n-1}\sum \limits_{i=1}^{n}{\left({\widehat{Y}}_{i}-\sum_{i=1}^{n}{\widehat{Y}}_{i}/n \right)}^{2}$$

The variance, SE, ME, CV, and 95% CI of the national-level estimates of sero-prevalence rate can be expressed in Eqs. (18–22).

18 $$Var\left(\widehat{P}\right)=\frac{Var\left(\widehat{\tau }\right)}{{M}^{2}}$$

19 $$SE\left(\widehat{P}\right)=\sqrt{\widehat{Var}\left(\widehat{P}\right)}$$

20 $$ME\left(\widehat{P}\right)=1.96 SE\left(\widehat{P}\right)$$

21 $$CV\left(\widehat{P}\right)=\frac{SE\left(\widehat{P}\right)}{\widehat{P}}\times 100$$

22 $$95 \% CI\left(\widehat{P}\right)=\widehat{P}\mp ME\left(\widehat{P}\right)$$

To derive the mathematical expressions, our approach makes the explicit assumptions that the vaccination program covers susceptible bovine population and QC passed NSP-free certified inactivated FMD vaccines are used, which are objectives of the NADCP. In the proposed methodology, state and national-level estimators are reported and are used to estimate the population level sero-prevalence parameters in India during the year 2008–2021.

### Structural equation modelling

Structural Equation Models (SEMs) are powerful multivariate tools extensively used in scientific investigations (viz., social sciences^38,39^, public health^40,41^, medical research^42^, etc.) to test and evaluate multivariate causal relationships^43^. However, there is a scarcity of SEM models in disease epidemiological research^44^ and thus Tu et al. urged epidemiologists to use the SEM models to understand the relationship among several epidemiological factors^45^. In this study, we used the SEM models first time in FMD research to understand the relationship between the sero-surveillance and field FMD outbreaks. With its strength as a statistical tool to analyse complex relationships among the variables and even to test causal relationships with non-experimental data, which makes this model as powerful tool in disease epidemiology. Further, the SEM can be seen as the combination of factor and regression analysis, which is used to analyse the structural relationship between measured variables and latent constructs. This method is preferred by the researchers because it estimates the multiple and interrelated dependence in a single analysis. Here, we have considered eight different SEM models in this study, which are briefly described in Table 1.

**Table 1:** Description of various Structural Equation Models (SEM) used in this study.

Sl. no.	Name	Models	Co-variates
1	Model 1	${p}_{t}={\alpha }_{0}+{\alpha }_{1}{O}_{t}+{\varepsilon }_{t}$	Current outbreak
2	Model 2	${p}_{t}={\alpha }_{0}+{\alpha }_{1}{O}_{t}+{{\alpha }_{2}{O}_{t-1}+\varepsilon }_{t}$	Current outbreak, outbreak (1 year lag)
3	Model 3	${p}_{t}={\alpha }_{0}+{\alpha }_{1}{O}_{t}+{{\alpha }_{2}{O}_{t-1}+{\alpha }_{3}{O}_{t-2}+\varepsilon }_{t}$	Current outbreak, outbreak (1 and 2 year lags)
4	Model 4	${p}_{t}={\alpha }_{0}+{\alpha }_{1}{O}_{t}+{{\alpha }_{2}{O}_{t-1}+{\beta }_{1}{p}_{t-1}+\varepsilon }_{t}$	Current outbreak, outbreak (1 year lag), previous positive rate
5	Model 5	${p}_{t}={\alpha }_{0}+{\alpha }_{1}{O}_{t}+{{\alpha }_{2}{O}_{t-1}+{\alpha }_{3}{O}_{t-2}+{\beta }_{1}{p}_{t-1}++\varepsilon }_{t}$	Current outbreak, outbreak (1 year lag), DIVA positive rate (1 year lag)
6	Model 6	${p}_{t}={\alpha }_{0}+{\alpha }_{1}{O}_{t}+{{\alpha }_{2}{O}_{t-1}+{\beta }_{1}{p}_{t-1}+{\beta }_{2}{p}_{t-2}+\varepsilon }_{t}$	Current outbreak, outbreak (1 year lag), DIVA positive rate (1 year lag), DIVA positive rate (2 year lags)
7	Model 7	${p}_{t}={\alpha }_{0}+{\alpha }_{1}{O}_{t}+{{\alpha }_{2}{O}_{t-1}+{\alpha }_{3}{O}_{t-2}+{\beta }_{1}{p}_{t-1}+{\beta }_{2}{p}_{t-2}+\varepsilon }_{t}$	Current outbreak, outbreak (1 year lag), outbreak (2 year lags), DIVA positive rate (1 year lag), DIVA positive rate (2 year lags)
8	Model 8	${p}_{t}={\alpha }_{0}+{\alpha }_{1}{O}_{t}+{{\alpha }_{2}{O}_{t-1}+{\alpha }_{3}{O}_{t-2}+{\beta }_{1}{p}_{t-1}+{\beta }_{2}{p}_{t-2}+{NADCP}_{t}+I({sample}_{t}+{pop}_{t})+\varepsilon }_{t}$	Current outbreak, outbreak (1 year lag), outbreak (2 year lags), DIVA positive rate (1 year lag), DIVA positive rate (2 year lags), NADCP program. Sample and population size

Model 1–8: Structural Equation Models; ${p}_{t}, {p}_{t-1}, {p}_{t-2}$: Estimated DIVA positive rates for *tth*, (*t-1*)*th*, and (*t-2*)*th* year respectively; ${O}_{t}, {O}_{t-1}, {O}_{t-2}$: Field FMDV outbreaks at *tth* , (*t-1*)*th*, and (*t-2*)*th* year respectively; ${\alpha }_{0}:$ intercept term; ${\alpha }_{i} \; {\text{and}} \; {\beta }_{i}$: co-efficient terms; $\varepsilon_{t}:$ error.

To implement the SEM, the *sem* R package (*ver*. 3.1-15)^46,47^ was executed and two-stage least square method^48^ was used to estimate the model parameters. Besides, for visualizing the results, we executed various functions implemented in R packages, namely ggplot2^49^, maptools^50^, ggmap^51^, and RColorBrewer^52^.

## Developed statistical software

**Figure 2:**
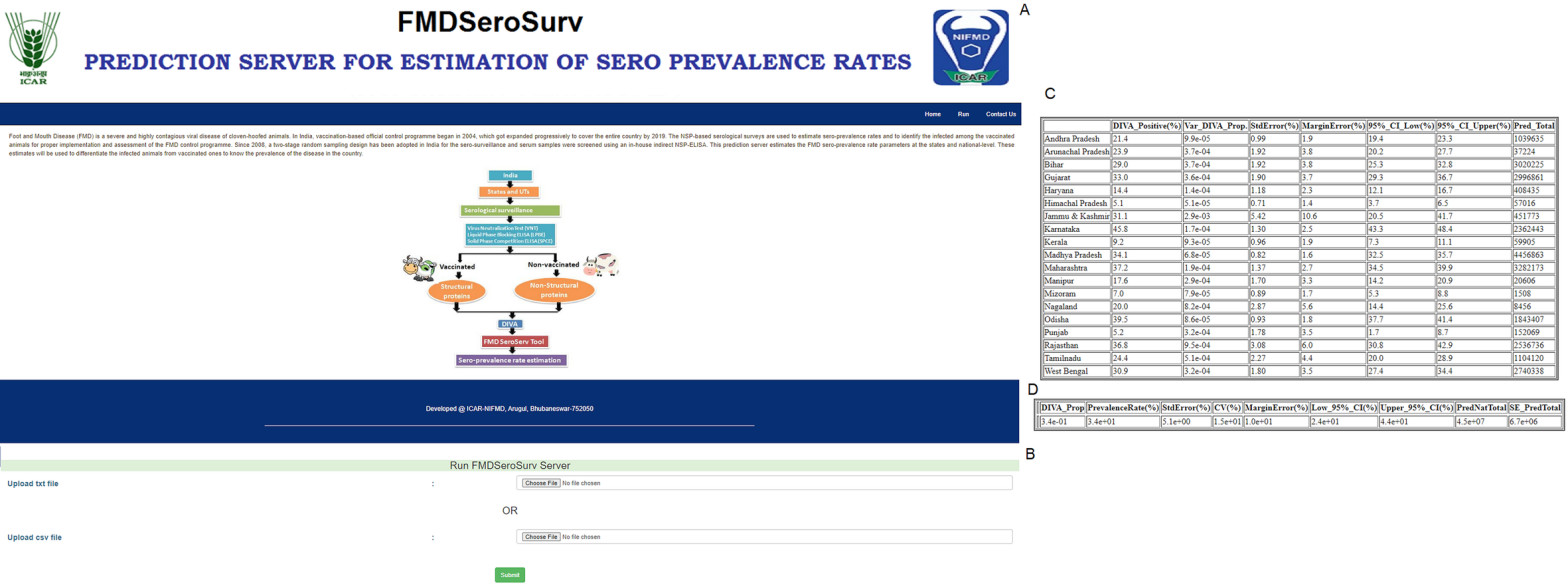
FMDSeroSurv web-application server. (**A**) Home page of the web-application. (**B**) Run page of the application. Here, the user will have to upload the data in .txt or .csv file for the analysis. (**C**) Output page represents the state-level estimate of sero-prevalence rates and related measures. (**D**) Output page represents the national-level estimate of sero-prevalence rates and related measures.

There is no publicly available web-application/tool for estimating sero-prevalence parameters for assessing FMDV infection in a susceptible animal population. So, we have implemented the developed approach in an online web-application “FMDSeroSurv” (https://nifmd-bbf.icar.gov.in/FMDSeroSurv) for estimation of sero-prevalence and its quality measures at state and national-level. The snapshots of the home page and analysis module are shown in Fig. 2A,B. Two result files are generated on the same page for state and national-level sero-prevalence estimations, shown in Fig. 2C,D. The user interface of the server was designed using HTML, JavaScript, CSS, and Bootstrap. The backend of the web application was developed using ASP.NET and R-program was integrated as statistical engine for execution of the proposed approach. The users can upload the data in standard format (e.g., .csv or .txt) and can also download the results (in .csv format into a zip file) from the server.

To facilitate off-line use of the developed methodology on users’ PC, an R-package, namely FMDSeroSurv (GPL-3.0 license), has been developed, which is freely available at https://github.com/sam-dfmd/FMDSeroSurv. This software provides functions to estimate the sero-prevalence rates along with various errors and population total at state and national level. The detailed user manual for the FMDSeroSurv R package and web-server is given in Supplementary Document S2 and https://nifmd-bbf.icar.gov.in/FMDSeroSurv respectively.

## Results

### State-wise estimation of sero-prevalence rates

**Figure 3:**
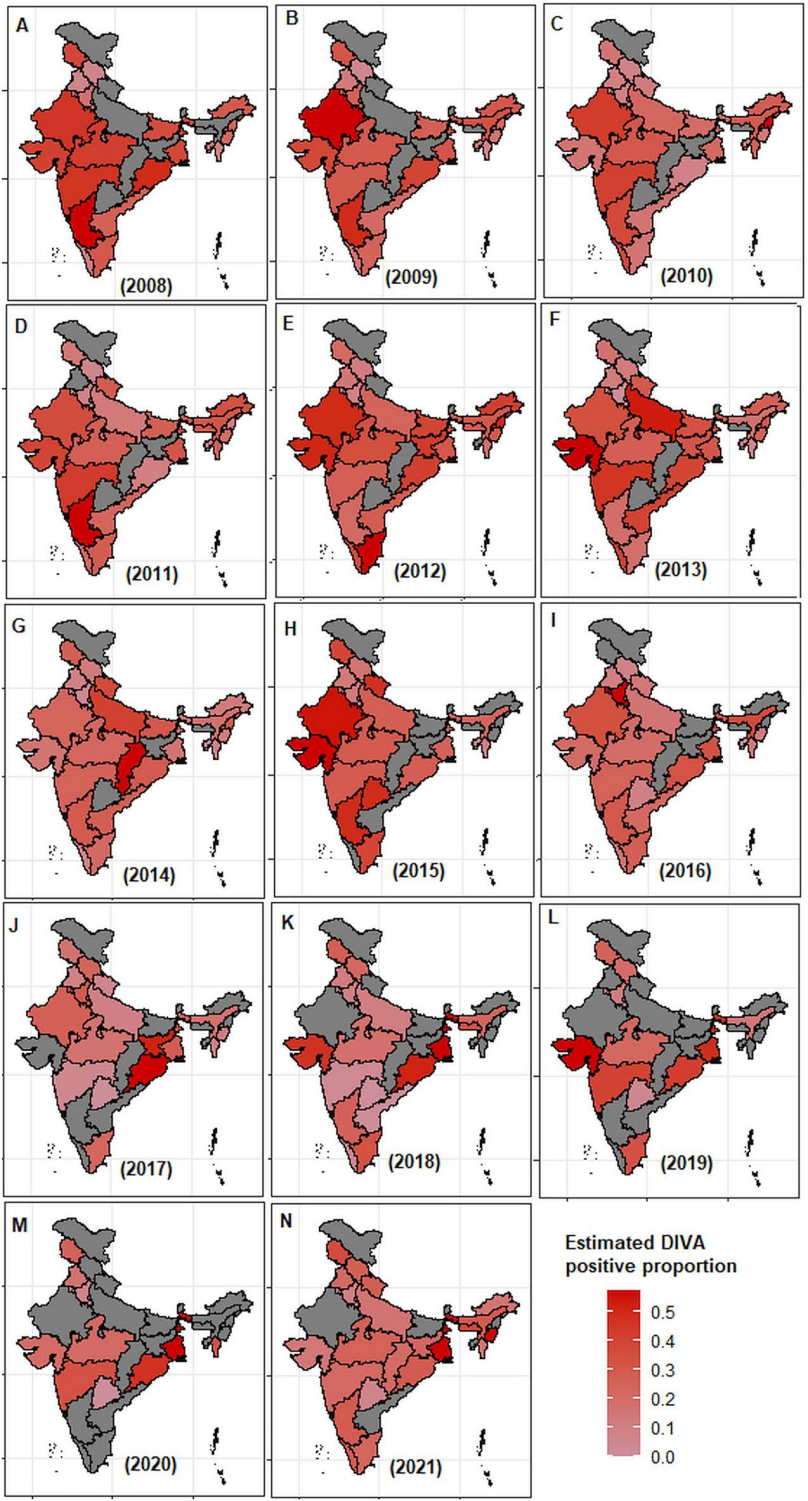
State-wise distribution of estimated sero-prevalence rates from the year 2008 to 2021. The estimated values of sero-prevalence rates are shown for various states covered during the year 2008 (**A**)–2021 (**N**). Higher the intensity of the red color means higher NSP sero-prevalence and *vice-versa*. The states shown in grey color are not covered in the sero-surveillance activities. A higher sero-prevalence estimated rate indicates more natural infection/virus in circulation of FMDV and *vice-versa*.

The developed methodology was applied to India’s FMDV sero-surveillance data for the year 2008–2021 to estimate the state-wise NSP sero-prevalence rates and the results are shown in Fig. 3. In 2008, the states including Karnataka, Odisha, Rajasthan, Gujarat, and West Bengal were found to have higher rates of NSP sero-prevalence compared to other states (Fig. 3A). Out of these, three states share international border with other countries. This means that these states had higher rates of FMDV infection (i.e. > 30%) among the susceptible population. For example, Karnataka had the highest sero-prevalence proportion of 0.458 (95% of CI [0.433, 0.484] and ME of 2.5%) with SE of 0.013 (Table S1). Further, a lower value of CV (i.e., 2.83%) was observed for the same state, indicating the higher precision of the sero-prevalence estimate compared to others (Table S1). The neighbouring states, including Punjab (95% CI [0.017, 0.087] with error: 3.5%), Himachal Pradesh (95% CI [0.037, 0.065] with error: 1.4%), and Haryana (95% CI [0.121, 0.167] with error: 2.3%) were found to have the lowest estimate of sero-prevalence rate with the highest CV values (34.52%, 13.92%, and 8.18% respectively) (Fig. 3A). This indicates that a higher degree of inconsistency among estimated sero-prevalence rates was observed for these states in 2008. Similar interpretations can be made for other states during 2008 from Fig. 3 and Table S1.

**Figure 4:**
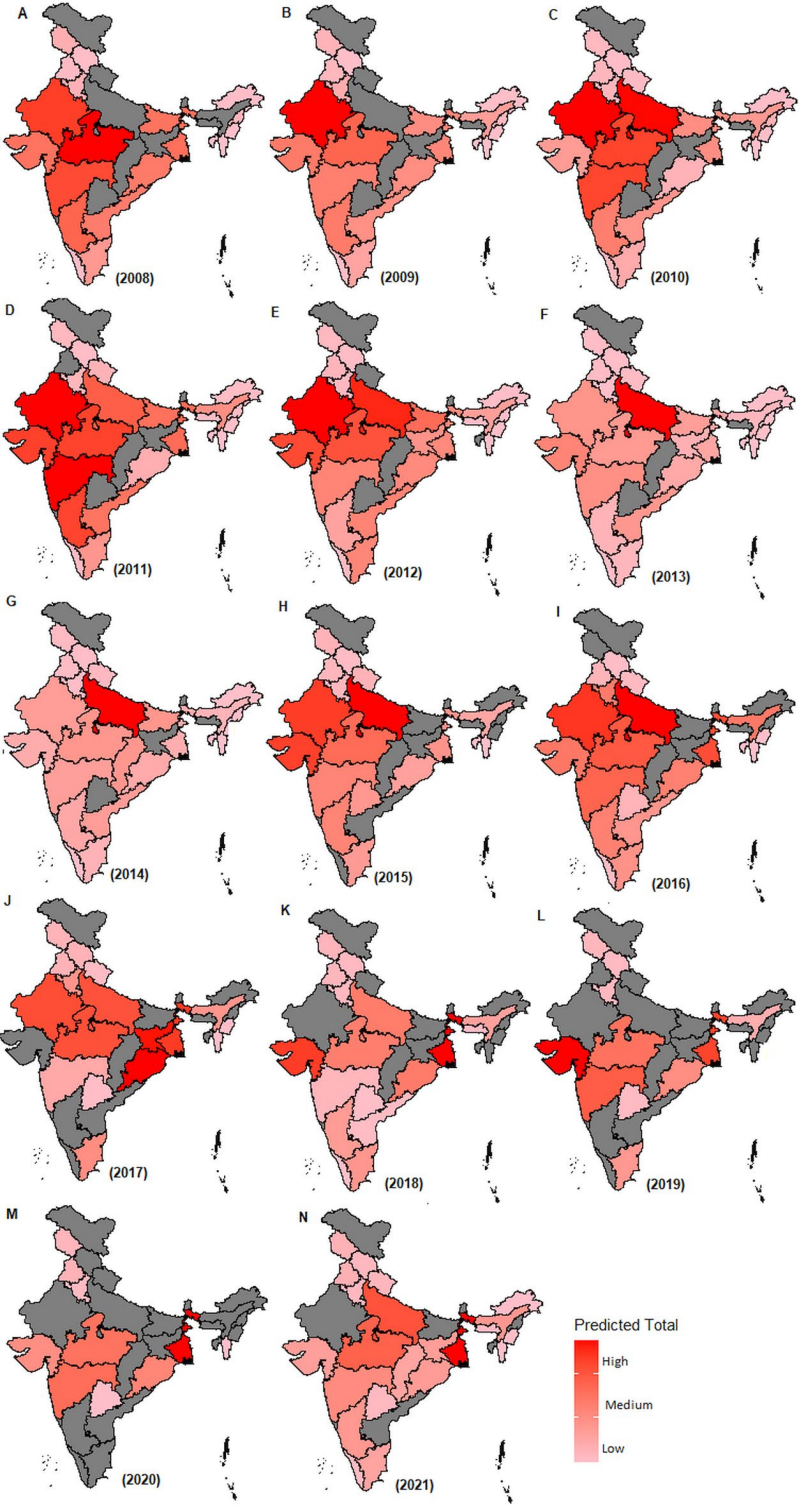
Distribution of predicted total DIVA positive animals during the year 2008–2021. The state-wise predicted total number of DIVA positive animals are shown for the year 2008 (**A**) to 2021 (**N**). Higher the intensity of red color indicates higher number of DIVA positive animals and *vice-versa*. The states shown in grey color are not covered in the sero-surveillance activities.

The results obtained from the analysis of FMDV sero-surveillance data for the years 2009–2021 are shown in Fig. 3, Supplementary Tables S2–S13, and Table 2. In 2021, for example, 96,497 sera samples were tested to assess the FMD sero-surveillance in India (Fig. S2). The states, including West Bengal and Manipur, were found to have highest NSP sero-prevalence rates, indicating higher FMDV infections in the susceptible bovine population (Fig. 3N, Table 2). These two states share international border with other countries, i.e., Bangladesh and Myanmar. Similar interpretations can be made for all the states during 2009–2020 (Fig. 3, Tables S2–S13). Further, the developed methodology was applied to estimate the population total (i.e., number of animals having history of infection) across various states during 2008–2021 and results are shown in Fig. 4 and Tables 2, S1–S13. For 2008 sero-surveillance, our method provided the highest estimate of NSP sero-positive animals for Madhya Pradesh, Rajasthan, and Maharashtra (Fig. 4 and Table S1). This could be due to their large susceptible bovine populations and highest rate of virus circulation. Similar interpretations can be made for other states from 2009 to 2021 (Fig. 4, Tables 2, S2–S13).

**Table 2:** Estimated values of DIVA positive rates for different states at the year 2021.

Sl. No.	STATE	${\widehat{P}}_{i}$	$SE({\widehat{P}}_{i})$	95% CI of ${\widehat{P}}_{i}$	$ME({\widehat{P}}_{i})$	$CV({\widehat{P}}_{i})$	${\widehat{Y}}_{i}$
1	Arunachal Pradesh	0.110	0.027	(0.057, 0.163)	0.053	24.44	15,247
2	Assam	0.212	0.005	(0.203, 0.221)	0.009	2.13	961,970
3	Chhattisgarh	0.150	0.006	(0.138, 0.161)	0.012	3.93	669,277
4	Gujarat	0.100	0.004	(0.092.0.108)	0.008	4.14	805,541
5	Haryana	0.068	0.003	(0.063, 0.074)	0.005	3.88	172,463
6	Himachal Pradesh	0.192	0.007	(0.179, 0.206)	0.013	3.50	190,442
7	Jammu & Kashmir	0.264	0.009	(0.246, 0.281)	0.018	3.39	340,737
8	Jharkhand	0.166	0.013	(0.141, 0.191)	0.025	7.81	835,176
9	Karnataka	0.207	0.009	(0.190, 0.225)	0.017	4.27	949,462
10	Kerala	0.211	0.004	(0.202, 0.220)	0.009	2.12	121,745
11	Madhya Pradesh	0.183	0.006	(0.172, 0.194)	0.011	3.03	2,124,385
12	Maharashtra	0.158	0.004	(0.151, 0.165)	0.007	2.25	1,239,090
13	Manipur	0.405	0.009	(0.389, 0.422)	0.017	2.11	42,284
14	Meghalaya	0.275	0.023	(0.230, 0.320)	0.045	8.36	101,170
15	Mizoram	0.122	0.017	(0.088, 0.156)	0.034	14.09	2337
16	Odisha	0.211	0.005	(0.200, 0.221)	0.010	2.48	873,189
17	Punjab	0.152	0.006	(0.140, 0.163)	0.011	3.78	396,954
18	Tamil Nadu	0.172	0.017	(0.140, 0.205)	0.032	9.60	692,238
19	Telangana	0.050	0.002	(0.045, 0.054)	0.004	4.59	168,877
20	Uttar Pradesh	0.117	0.003	(0.110, 0.123)	0.006	2.84	2,425,666
21	Uttarakhand	0.200	0.006	(0.188, 0.213)	0.012	3.14	217,798
22	West Bengal	0.410	0.017	(0.376, 0.444)	0.034	4.22	3,231,276

${\widehat{P}}_{i}$: Estimated value of DIVA positivity rate; $SE({\widehat{P}}_{i})$: Estimated value of standard error of ${\widehat{P}}_{i}$; 95% CI of ${\widehat{P}}_{i}$: 95% confidence interval of ${\widehat{P}}_{i}$; $ME({\widehat{P}}_{i})$: Estimated value of error margin for ${\widehat{P}}_{i}$; $CV({\widehat{P}}_{i})$: co-efficient of variation of ${\widehat{P}}_{i}$; ${\widehat{Y}}_{i}$: estimated number of DIVA positive animals in each state.

### National-level sero-prevalence rate estimation

**Figure 5:**
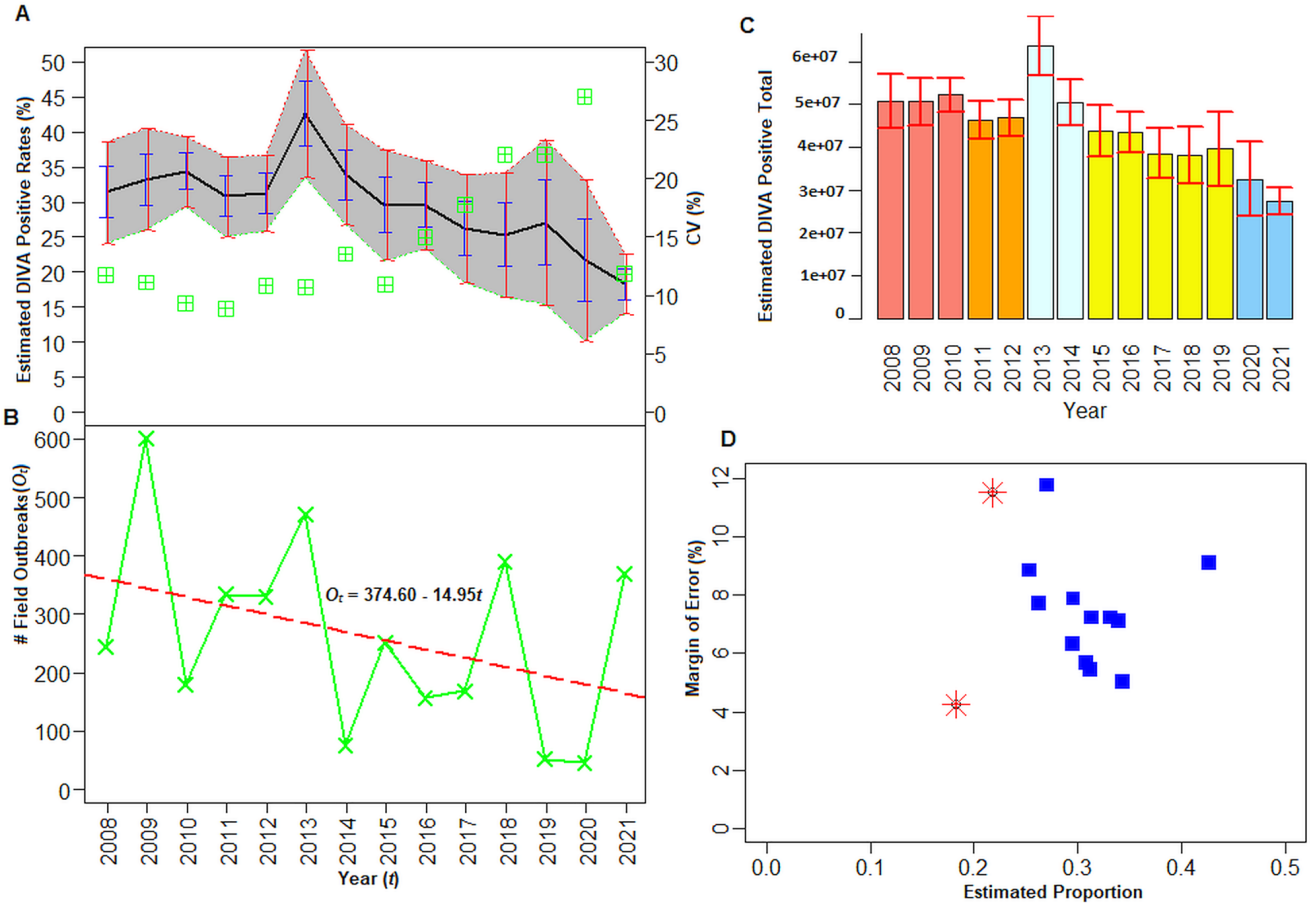
National-level (India) estimation of FMDV NSP sero-prevalence. (**A**) Year-wise distribution of estimated sero-prevalence rates and their computed confidence intervals, standard and margin of errors are shown. X-axis represents the years. Y1-axis represents the estimated sero-prevalence rates. Y2-axis represents the estimated co-efficient of variations. Grey color indicates the 95% confidence region of the national-level sero-prevalence estimates. Red color line represents upper and lower bound of 95% confidence intervals. The blue and red color bars indicate the standard and marginal errors of the estimates respectively. The green color (cross-squares) boxes show the co-efficient of variation of the national-level sero-prevalence estimates. (**B**) Distribution of the field FMD outbreaks during the year 2008–2021. X-axis: Years; Y-axis: FMD outbreaks. Red color line represents the fitted regression line. (**C**) Bar diagram for the estimated total of DIVA positive animals over the years (2008–2021). X-axis: Years and Y-axis: Estimated DIVA positive numbers. Red color bars represent the standard errors. (**D**) Relation between estimated DIVA positive rates and their margin of error. X-axis: DIVA positive rate; Y-axis: percentage of margin of errors. Red color star points represent the relationship during COVID-19 period.

The developed methodology was applied to India’s FMD sero-surveillance data from 2008 to 2021 to compute the national-level estimates of NSP sero-prevalence. The results are shown in Fig. 5 and Supplementary Table S14. The year-wise distributions of estimated sero-prevalence rates are shown, along with their 95% confidence regions, SE, and ME in Fig. 5. In 2008, our statistical approach computed the national-level estimate of the sero-prevalence rate as 31.40% (with 95% CI [24.19, 38.66]) with a 7.23% ME (Fig. 5, Table S14). Through our developed approach, we also provided an estimate of infected animals among the susceptible population in 2008 (Fig. 5C). This estimate is associated with higher error, as it is difficult to get the actual size of the susceptible bovine population in India. Similar interpretations can be made for the remaining years (Fig. 5).

Through the graphical analysis, we observed relatively static trend for sero-prevalence rates till 2012, then sharp spike in 2013 and after that a downward trend was observed with minimal value in 2021 (Fig. 5A). The national-level FMD sero-prevalence rates were estimated as 31.4% (CV: 11.74%), 33.2% (CV: 11.11%), and 34.4% (CV: 9.37%) for the year 2008, 2009, and 2010 respectively (Fig. 5A). Post 2010, the estimates were found to be relatively lesser compared to 2008–2010, which might be due to higher reported outbreaks and narrow coverage of sero-surveillance during the latter period (Figs. 5B, S2). In 2013, the highest sero-prevalence rate was observed in India, which might be due to higher number of outbreaks during that period (Fig. 5A,B). The sero-prevalence rates gradually declined post 2013, but higher CV and error rates were observed in 2020 (Fig. 5A), indicating disruption in sero-surveillance and diagnosis activities might be due to the imposed lock down during COVID19. For instance in 2020, the estimated sero-prevalence rate was found to be 21.75% with higher error (11.51%) and CV (27%). Furthermore, the sero-prevalence rate in 2021 was estimated at 18.27% (95% CI [14.02%, 22.52%]) with ME of 4.25% (Table S14). The lowest value of the estimate, with minimum CV and error, indicates the India’s ability to estimate sero-prevalence with higher confidence and broader sero-surveillance system in place. The overall declining trends in sero-prevalence rates indicated a lower rate of FMDV infections among the susceptible animals over the years.

The distribution of the field FMD outbreaks over the years is also shown in Fig. 5B. As of now, a total of 3675 outbreaks have been reported over a period of 14 years (2008–2021), with substantial heterogeneity in the temporal occurrence of outbreaks (Fig. 5B). Further, the highest FMD outbreaks were reported for the year 2009, followed by 2013 and 2018 (Fig. 5B). A linear regression line was fitted to the temporal distribution of the outbreak data, and the results are shown in Fig. 5. The fitted regression line (negative value of the slope) indicated a downward trend in FMD outbreaks over the years (Fig. 5B). In other words, downward trends were observed for both the temporal distributions of sero-prevalence rates and field outbreaks (Fig. 5A,B), which might indicate the success of vaccination programs in India.

### Relationship between NSP-serology and field outbreaks

**Figure 6:**
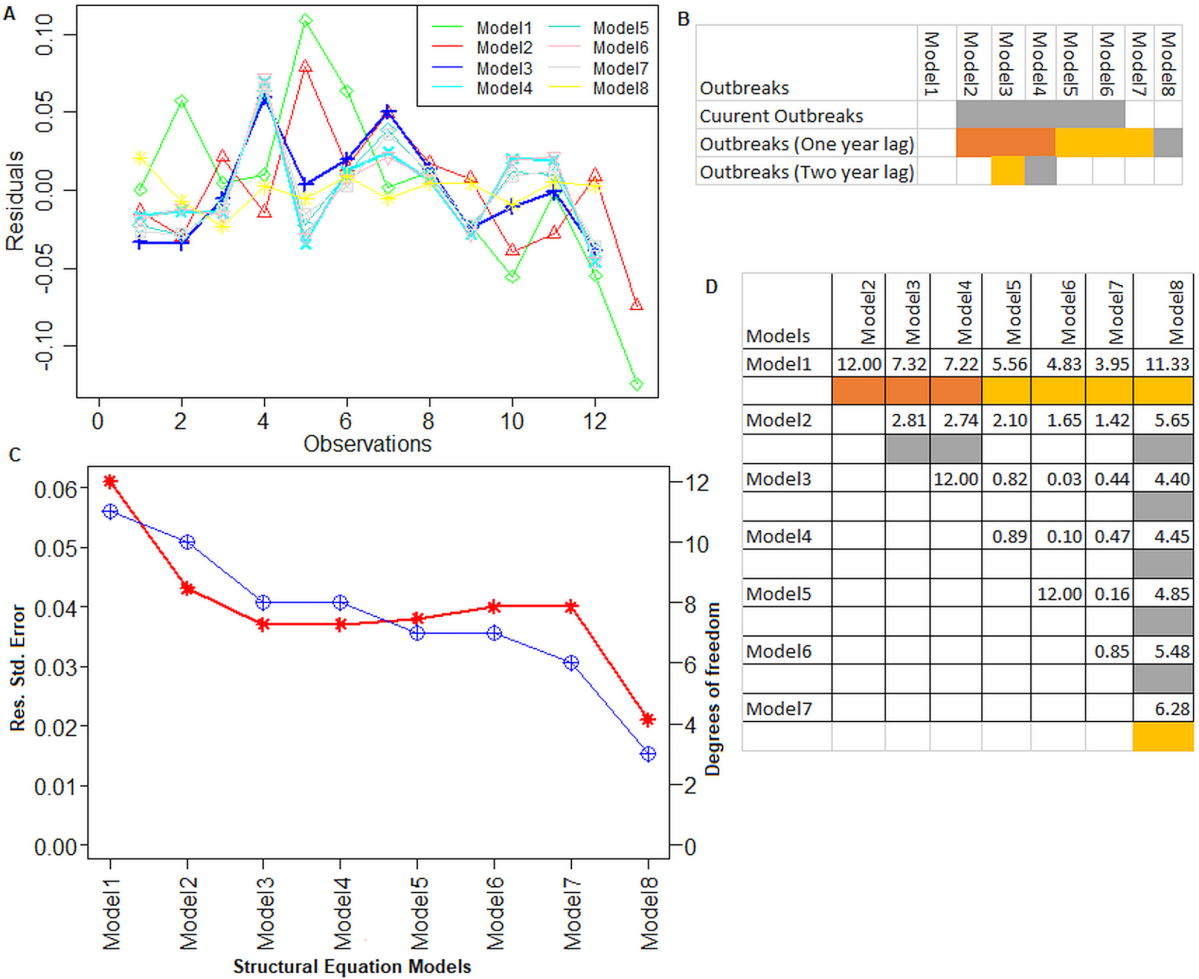
Relationship among estimated sero-prevalence rate and FMD outbreaks through structural equation modelling. (**A**) Performances of eight different SEMs are shown. X-axis: observations. Y-axis: values of residuals. Residual plots for the SEMs are shown in different colors. (**B**) Significance of current year and previous year outbreaks on estimated sero-prevalence rate is shown through SEMs. Yellow, orange and grey color shows the statistical significance of values at 10%, 5%, and 1% level of significances. (**C**) SEMs *vs.* residual standard error and error degrees of freedom plot. X-axis represents the models. Y-axis represents residual standard error. Y1 axis represents error degrees of freedom. Blue color points show the distribution of error degrees of freedom. Red color points show the distribution of residual standard errors. (**D**) Relationship among the SEMs. Yellow, orange and grey colors show the statistical significance of test statistic(s) at 10%, 5%, and 1% level of significances.

We used the SEM approach to investigate the relationship between estimated sero-prevalence rates and the field FMD outbreaks reported in India. Next, we built eight different structural models to model the casual relation of the rate of virus circulation in recent times with field FMD outbreaks and other latent variables. The results from the SEM analysis are shown in Table 3 and Fig. 6. The covariates, including outbreaks, previous NSP sero-prevalence rates, vaccination status, sample, and population size, were considered in the SEM analysis (Table 3). The residuals obtained from each of the SEMs are plotted in Fig. 6A. The results indicated that Model 3 performed well in terms of lower residuals compared to other models (Fig. 6A). Alternatively, Model 3 followed by Model 4 provided best fit to the data, showing good relationship between the estimated sero-prevalence rates with outbreaks and other covariates (Fig. 6A).

**Table 3:** Co-efficient estimation and analysis of variance for various structural equation models.

Sources of variation	Co-efficient	Std. error	Test statistic	p-value	Sig.
Model 1 (0.061)					
(Intercept)	0.267	0.032	8.27	4.7E−06	****
Outbreaks	0.0001	0.0001	1.0288	0.326	
Model 2 (0.043)					
(Intercept)	0.188	0.033	5.778	0.0002	****
Outbreaks	0.0002	0.0001	2.0692	0.065	*
Outbreaks (1 year lag)	0.0003	0.0001	3.4643	0.006	***
Model 3 (0.037)					
(Intercept)	0.1319	0.037	3.5681	0.007	***
Outbreaks	0.0002	0.0001	2.2807	0.052	*
Outbreaks (1 year lag)	0.0003	0.0001	4.3347	0.002	***
Outbreaks (2 year lag)	0.0002	0.0001	2.3234	0.049	**
Model 4 (0.037)					
(Intercept)	0.0179	0.0749	0.2386	0.817	
Outbreaks	0.0002	0.0001	2.4147	0.042	*
Outbreaks (1 year lag)	0.0002	0.0001	2.4132	0.012	***
DIVA prop (1 year lag)	0.5975	0.249	2.3991	0.043	*
Model 5 (0.038)					
(Intercept)	0.052	0.087	0.600	0.567	
Outbreaks	0.000	0.000	2.349	0.051	*
Outbreaks (1 year lag)	0.000	0.000	2.423	0.046	**
DIVA prop (1 year lag)	0.379	0.372	1.019	0.342	
Outbreaks (1 year lag)	0.000	0.000	0.796	0.452	
Model 6 (0.040)					
(Intercept)	0.012	0.089	0.133	0.898	
Outbreaks	0.000	0.000	2.051	0.079	*
Outbreaks (1 year lag)	0.000	0.000	2.265	0.048	**
DIVA prop (1 year lag)	0.543	0.337	1.611	0.151	
DIVA prop (2 year lag)	0.072	0.339	0.213	0.838	
Model 7 (0.040)					
(Intercept)	0.045	0.099	0.454	0.666	
Outbreaks	0.000	0.000	1.936	0.101	
Outbreaks (1 year lag)	0.000	0.000	2.303	0.061	*
DIVA prop (1 year lag)	0.279	0.469	0.594	0.574	
DIVA prop (2 year lag)	0.111	0.346	0.320	0.760	
Outbreaks (1 year lag)	0.000	0.000	0.820	0.444	
Model 8 (0.021)					
(Intercept)	0.268	75,209.6	0.0001	0.997	
Outbreaks	0.002	53.0	0.0001	0.15	
Outbreaks (1 year lag)	0.001	38.4	0.0001	0.079	*
DIVA prop (1 year lag)	− 0.091	314,502.7	0.0001	0.956	
Outbreaks (1 year lag)	0.002	65.1	0.0001	0.978	
Vaccination program	0.019	19,520.9	0.0001	0.999	
Total sample	0.003	0.2	0.0001	0.997	
Positive sample	0.001	1.0	0.0001	0.992	
DIVA prop (2 year lag)	− 0.110	37,535.8	0.0001	0.979	

Model 1–8: Structural Equation Models (SEM); Std. Error: Standard error; sig.: Significance levels; *, **, ***, ****: values significant at 10%, 5%, 1%, and 0.1% level of significances; *p-value*: statistical significance values of co-variates included in the SEM; values in ():Std. Error of residuals for each SEM.

The residual standard error and error degrees of freedom of the models are shown in Fig. 6C. The Model 3 provided the lowest residual error with relatively sufficient error degrees of freedom compared to other models (Fig. 6C), which is at par with the Model 4. It is interesting to note that the Model 8 provided the least residual standard error along with lowest degrees of freedom for estimating it (Fig. 6C). However, it also required large number of covariates to model the relationship, which may not be readily available (Tables 1, 3). Hence, both the comparative analyses of the models indicated that Model 3 is suitable to study the relationship between the sero-prevalence rates for FMD sero-surveillance and the field outbreaks in India. Furthermore, we also tested whether the model’s performance was significantly different or not by executing the *anova* function in R. The results in terms of F-statistics and *p-values* are shown in Fig. 6D. The performance of Model 1 was highly similar to that of the other remaining models (Models 2–8) (Fig. 6D). It is due to the fact that Model 1 is the simplest model and has only one covariate, which was also included in other models (Fig. 6D). Further, the more complex model, i.e., Model 8, is significantly better at capturing the relationships between the estimated sero-prevalence rates and the covariates (Fig. 6C,D). However, it is quite complex and requires a lot of information for model fitting compared to other models (Table 1).

Next, we tried to establish the relationship between current sero-prevalence rates and past FMD outbreaks. For this purpose, we used 8 different SEMs and the results are shown in Fig. 6C. The results indicated that the estimated sero-prevalence rates (for a particular year) was found to be (statistically) significantly associated (at 10% level of significance) with same year’s FMD outbreak for SEMs 2–7. The sero-prevalence rates are (statistically) significantly (at 1% level of significance for models 2–4; 5% level of significance for models 5–7; at 10% level of significance for model 8) depend on the past FMD outbreaks (Fig. 6C). Broadly, at higher levels (i.e., 5% and 1%) of significance, the current year’s estimated sero-prevalence rates were found to significantly depend on the preceding years’ infection. Through the best performing model, i.e., Model 3, we observed that the current sero-prevalence rate depends on current outbreaks along with outbreaks of previous 2 years (Fig. 6C).

### Relationship among FMDV sero-surveillance parameters

The relationships among the estimated parameters, including sero-prevalence rates their SE, ME and CV, computed through the developed approach, are shown in Figs. 5D, S7–S10. The estimated sero-prevalence rate in India increased sharply in 2013 and after that gradually declined until 2021 (Fig. S7). This declining trend of NSP prevalence could be due to downward trend of the FMD outbreaks (Fig. 5A,B). The trend in distribution of the CV values of the NSP sero-prevalence rates was found to be increasing up to 2020 and after that it declined steeply (Fig. S7). For the years 2019 and 2020, a lower NSP-prevalence rate was observed in India compared to previous years corroborating with decline in the number of outbreaks but with higher rates of error and CV (Figs. 5 and S7). During this period, relatively lesser numbers of serum samples (i.e., narrow coverage of the surveillance) were collected for testing (Figs. S1, S2). It is pertinent to note that lower rates of NSP-prevalence along with lower error rates were observed for the year 2021, followed by 2017 and 2018 (Table S14, Figs. 5D, S8–S10). Further, the sero-prevalence estimates with lower CVs were observed to have lower rates of estimation errors (Fig. S9).

## Discussion

The hindrances to the control of FMD in India are diverse and include several socio-ecological factors, viz*.* unrestricted movement of animals, porous inter-state borders, asymptomatic infections, unavailability of timely and quality vaccines, timely high density vaccination, etc. Due to highly contagious nature of the virus and non-sterile immunity after vaccination, both vaccinated and non-vaccinated animals are infected in a situation of sub-optimal herd immunity. Estimating the FMD sero-prevalence rate is crucial for monitoring the vaccination programme through detecting NSP-Abs of the past and recent infections. There is no provision in the standard sero-surveillance activity in India to provide a population-level estimation of sero-prevalence rates. This observation is true for other countries as well, where FMD is endemic^29,33–36^. In simple words, no statistical approach and computational tool are available till date for estimating the state and national-level FMDV prevalence rate in India. Thus, we presented a statistical approach and computational tools for this purpose and applied them to analyse India’s FMD sero-surveillance data during the year 2008–2021. Furthermore, our approach makes certain assumptions about the FMD sero-surveillance process: (*i*) vaccination covers the susceptible bovine population in the country; (*ii*) QC passed NSP-free FMD vaccines are administered. These assumptions are realistic, which are the basic objectives of the disease control programs including the NADCP.

Here, we reported unbiased estimators for estimating the population NSP sero-prevalence rate and total number of animals having history of infection. The expressions for computing variance of the estimator and its estimated value at state and national-levels were reported. For instance, the estimated variance of the national-level NSP sero-prevalence rate captured the variance between the states and variance within the states. The variance of different estimators along with other measures could be used to assess the quality of the estimates. Further, the animal census data, usually collected in every 5 years, were well utilized in the developed approach to estimate various parameters. The developed approach was implemented in two user-friendly tools for better execution, which are freely available. The estimated parameters can be used to track the infection at the population-level under vaccination and further formulate the policy for declaring disease free zones.

To control FMD, a mass vaccination programme with inactivated trivalent vaccines has been practised in India since the year 2004 starting from 54 districts to whole country in the year 2019. Earlier, India employed a tetravalent FMD vaccine (O, A, C, and Asia1), with different vaccine strains being used by the vaccine manufacturers. Since October 2003, the vaccine formulation has not included serotype C because it has not been detected since 1995. Instead, a trivalent vaccine formulation (O, A, and Asia1) with a uniform vaccine strain policy has been implemented. Next, it is important to estimate state-wise NSP-prevalence rates to study the impact of the vaccination. Through the developed approach, sero-prevalence rate estimates were obtained for the selected states during the year 2008–2021. These estimates were very crucial to know the circulation of the virus in the states under vaccinated condition and thus would help in devising control strategy at the local-level.

The estimated sero-prevalence rates of the bordering states (e.g., West Bengal, Manipur, Rajasthan, etc., i.e., states that share international border with other countries) were observed to be higher compared to others. This might be due to illegal transboundary movements of animals across the border, which increases the chance of potential transboundary introductions of novel FMDV into India through the border states. This observation is in coherent with previous studies that indicated the transportation of cattle and buffaloes from India to Malaysia through Myanmar and Vietnam (all pool 1 countries), which might lead to the dissemination of FMDV^16,53,54^.

Through the developed methodology, we presented the temporal distribution of the NSP sero-prevalence estimates in India to assess the FMDV infections among the susceptible bovine population. The relatively high sero-prevalence rate in 2008–2010 might be attributed to higher FMDV infection, low vaccination coverage and small sample size, as only 54 districts were covered under the FMDCP until 2010. The low intensity vaccination was reflected in the relatively higher NSP sero-prevalence rates in subsequent 2 years. In addition, spikes were observed for the years 2013–2014 due to higher outbreaks or usage of low quality of vaccines. After that, sero-prevalence rates gradually decreased as more districts were covered under the FMDCP and has achieved its lowest value (with minimal error and CV) for the year 2021. This might be due to the expansion of mass vaccination programme to cover all the susceptible animals against FMD under the NADCP by the Government of India after 2019. During the COVID-19, lower rates of sero-prevalence (with higher error and CV) were observed, which could be due to minimal social activities and restrictions on animal and human movements. Besides, higher error rate associated with the estimator indicated lower sample size and irregularities in sample collection, narrow surveillance, and restricted movement of survey professionals due to the imposed lock down. In 2021, lower NSP sero-prevalence rate (with lower CV and errors) was observed, showing a lesser FMDV outbreak in India with a strong and robust system of sero-surveillance and an efficient control mechanism. Ideally, a lower estimated sero-prevalence rate with lower CV/error rate is usually preferred, which indicates implementation of stricter surveillance and adoption of comprehensive sampling. The overall trend in distribution of sero-prevalence rates has been gradually declining over the years. The declining NSP prevalence among the susceptible animal population is considered as one of the essential success indicators of the FMDCP and is an important pre-requisite for official endorsement by the WOAH. This finding was well supported by the declining trends in the temporal distribution of FMD outbreaks reported in India. To achieve this, the sustained efforts of the Government of India and use of better diagnostics supported by precautionary measures taken by FMD research institute and state FMD centres could not be ruled out. If this trend continues, India will achieve FMD-free status with vaccination by 2030 from its third stage in progressive control pathway (i.e., outcome-oriented guidelines by the WOAH)^15^.

Through SEM analysis, we established that the sero-surveillance results are highly dependent on previous natural infections, which amounts to the production and persistence of NSP-Abs in the susceptible animals. It could be due to during active viral replication following FMDV infection; arrays of NSPs are produced that elicit anti-NSP antibodies. The past or recent infections can be detected by induced antibodies against FMDV NSP. In endemic settings with practising vaccination, the NSP serological tests are widely used to support the sero-surveillance activities that assess the prevalence of FMDV infection in susceptible animals^31,34,55–57^ and wild life^58^, as structural protein based tests are constrained for DIVA. It is well known that the samples detected positive in NSP-ELISA are mostly due to previous FMDV infections^16^. In simple words, we established that the NSP sero-prevalence rate is highly related to infections up to 2 years, which might indicate the NSP-Abs persist at detectable level during post-infection depending on the level of virus replication and strength of NSP-Abs induced, often lasting up to 2 years. For instance, we observed that sero-prevalence rate for 2021 significantly depends on the FMD outbreaks of 2020 and 2019. Here, we could not establish any significant link between the mass vaccination programme and estimated sero-prevalence rate due to lack of sufficient data. In India, the NADCP was started on September 2019 leaving only two data points (2020 and 2021) to show any impact in the modeling. Besides, we identified the best model to link the current sero-prevalence rate with previous infections with a proper statistical basis at the country-level.

## Conclusion

In FMD-endemic countries including India, the NSP sero-prevalence rate is a crucial indicator for impact assessment of the disease control programme. Thus its estimation is crucial in formulating the FMD control policies. Therefore, we proposed a novel statistical approach for sero-surveillance parameter estimation. This approach can perform analysis including estimating the NSP sero-prevalence rates at population-level along with related measures including various errors, CI of estimator, predicted total number of NSP positive animals, etc. Here, we provided all the background statistical theory and real survey data applications, along with interpretations of the obtained results. Besides, two user-friendly tools were reported based on the proposed approach. These tools are freely available to the users for data analysis without delving deeper into the methodology. This study provides valuable tools for the FMD researchers to get real-time estimates of sero-prevalence at various levels to implement control programmes. Our study established the overall relationship between the estimated sero-prevalence rates and field FMD outbreaks, which will provide valuable inputs to plan future studies including disease forecast model building. Though there might be a chance of under reporting of FMD incidences, so researchers may think of developing statistical models for prediction of outbreak incidences at the population-level. In future, these estimates along with vaccination rates (determined at population-level) and transboundary movement of animals may be considered in the SEM models to study the relationship between sero-prevalence and other variables including vaccination, outbreak, transboundary movement, etc. Besides, overall downward trends in the distributions of sero-prevalence rate and outbreak were observed in India over the years, which might be due to the implementation of disease control measures and robust sero-surveillance system. The developed method, models, and tools can be extended to sero-surveillance of other viral diseases in humans, plants, and animals.

### Supplementary Information

Supplementary Information.

## Data Availability

The secondary datasets used in this study are available in https://github.com/sam-dfmd/SeroSurveillance. The source R codes of the web-application are also provided in https://github.com/sam-dfmd/FMDSeroSurv.
