# Supplementary material for: Estimation of foot-and-mouth disease virus sero-prevalence rates using novel computational approach for the susceptible bovine population in India during the period 2008–2021
Source: Sci Rep. 2023 Dec 19;13:22583. doi: 10.1038/s41598-023-48459-w (PMC10730831; doi:10.1038/s41598-023-48459-w)
Supplement: Supplementary file 1 — Supplementary Information. [file 41598_2023_48459_MOESM1_ESM.docx]

**Supplementary Materials**

**Of**

Estimation of foot-and-mouth disease virus sero-prevalence rates using novel computational approach for the susceptible bovine population in India during the period 2008–2021

Samarendra Das^1,*^, Soumen Pal^2^, Sagar Sangam Rautaray^1^, Jajati K. Mohapatra^1^, Saravanan Subramaniam^1^, Manoranjan Rout^1^, Shesh N. Rai^3,*^, Rabindra Prasad Singh^1,*^

^1^ICAR-National Institute on Foot and Mouth Disease, International Centre for Foot and Mouth Disease, Aragul, Bhubaneswar 752050, Odisha, India

^2^Division of Computer Application, ICAR-Indian Agricultural Statistics Research Institute, New Delhi-110012, India

^3^College of Medicine, University of Cincinnati, Cincinnati-45267, OH, USA

**Authors’ email addresses**:

SD: [samarendra.das@icar.gov.in](mailto:samarendra.das@icar.gov.in)

SP: [soumen.pal@icar.gov.in](mailto:soumen.pal@icar.gov.in)

SSR: [sagarsangam28@gmail.com](mailto:sagarsangam28@gmail.com)

JKM: [jajati.mohapatra@icar.gov.in](mailto:jajati.mohapatra@icar.gov.in)

SS: [s.subramaniam@icar.gov.in](mailto:s.subramaniam@icar.gov.in)

MR: [manoranjan.rout@icar.gov.in](mailto:manoranjan.rout@icar.gov.in)

SNR: [raise@ucmail.uc.edu](mailto:raise@ucmail.uc.edu)

RPS: [rabindra.singh@icar.gov.in](mailto:rabindra.singh@icar.gov.in)

*To whom correspondence should be addressed- email: [samarendra.das@icar.gov.in](mailto:samarendra.das@icar.gov.in), [rabindra.singh@icar.gov.in](mailto:rabindra.singh@icar.gov.in), [raise@ucmail.uc.edu](mailto:raise@ucmail.uc.edu).

**Supplementary Document S1**: Proof of the FMDSeroSurv methodology.

Population variance for i^th^ state in t^th^ year:

$$S_{it}^{2}=\frac{1}{M_{it}-1}\sum_{j=1}^{M_{it}} y_{ijt}^{2}-M_{it}Ȳ_{it}^{2}$$

$$=\frac{1}{M_{it}-1}[M_{it}P_{it}-M_{it}P_{it}^{2}]$$

$$=\frac{M_{it}}{M_{it}-1}P_{it}(1-P_{it})$$

$$=\frac{M_{it}(P_{it}Q_{it})}{M_{it}-1}$$

Sample variance for i^th^ state in t^th^ year:

$$s_{it}^{2}=\frac{1}{m_{it}-1}\sum_{j=1}^{m_{it}} {(y_{ijt}-ȳ_{it})}^{2}$$

$$=\frac{1}{m_{it}-1}\left( \sum y_{it}^{2}-m_{it}ȳ_{it}^{2} \right)$$

$$=\frac{1}{m_{it}-1}\left( m_{it}p_{it}-m_{it}p_{it}^{2} \right)=\frac{m_{it}}{m_{it}-1}p_{it}q_{it}$$

[$where, q_{it}=1-p_{it}]$

As samples are drawn following SRSWOR procedure,

1. Sample proportion is the unbiased estimator for population proportion showing FMDV antibody titre.

Mathematically,

$$E\left( p_{it} \right)=E\left( ȳ_{it} \right)=Ȳ_{it}=P_{it}$$

Means, $p_{it}$ is the unbiased estimator for $P_{it}$.

i.e. ${P̂}_{it}=p_{it}$

$${Â}_{it}=M_{it}{P̂}_{it}$$

1. Variance of the estimator:

$$V\left( p_{it} \right)=V(ȳ_{it})$$

$$=\frac{{M_{it}-m}_{it}}{M_{it}m_{it}}S^{2}$$

$$=\frac{{M_{it}-m}_{it}}{M_{it}m_{it}}X\frac{M_{it}P_{it}Q_{it}}{M_{it}-1}$$

$$=\frac{{M_{it}-m}_{it}}{m_{it}(M_{it}-1)}P_{it}Q_{it}$$

$$V\left( p_{it} \right)=\frac{{M_{it}-m}_{it}}{m_{it}(M_{it}-1)}P_{it}Q_{it}$$

$$\Rightarrow$$

1. As the population proportions, i.e., $P_{it}{,Q}_{it}$ are unknown these need to be estimated from samples. In other words, the estimate of $V(p_{it})$ can be expressed as:

$$\hat{V}\left( p_{it} \right)=v\hat{a}r\left( ȳ_{it} \right)=\frac{{M_{it}-m}_{it}}{M_{it}m_{it}}s^{2}$$

$$=\frac{{M_{it}-m}_{it}}{M_{it}m_{it}}X\frac{m_{it}}{m_{it}-1}p_{it}q_{it}$$

$$=\frac{{M_{it}-m}_{it}}{M_{it}(m_{it}-1)}p_{it}q_{it}$$

$$v\hat{a}r\left( p_{it} \right)=\frac{{M_{it}-m}_{it}}{M_{it}(m_{it}-1)}p_{it}q_{it}$$

^⇒^

**Supplementary Tables**: State-level estimates of the DIVA rate and other related parameters.

The developed FMDSeroSurv methodology was applied to FMDV sero-surveillance data of India for the year 2008-21 to estimate the state-level DIVA rate and other parameters. The results are shown in Table S1-S13 for the Year 2008-20 and Table 2 (main text) for the year 2021.

**Table S1**. State-wise estimated values of DIVA positive rates for the year 2008.

| **Sl. No.** | **STATE** | ${\hat{\boldsymbol{P}}}_{\boldsymbol{i}}$ | $\boldsymbol{SE(}{\hat{\boldsymbol{P}}}_{\boldsymbol{i}}\boldsymbol{)}$ | **95% CI of** ${\hat{\boldsymbol{P}}}_{\boldsymbol{i}}$ | $\boldsymbol{ME(}{\hat{\boldsymbol{P}}}_{\boldsymbol{i}}\boldsymbol{)}$ | $\boldsymbol{CV(}{\hat{\boldsymbol{P}}}_{\boldsymbol{i}}\boldsymbol{)}$ | ${\hat{\boldsymbol{Y}}}_{\boldsymbol{i}}$ |
| --- | --- | --- | --- | --- | --- | --- | --- |
| 1 | Andhra Pradesh | 0.214 | 0.010 | (0.194, 0.233) | 0.019 | 4.66 | 5047781 |
| 2 | Arunachal Pradesh | 0.239 | 0.019 | (0.202, 0.277) | 0.038 | 8.033 | 119375 |
| 3 | Bihar | 0.290 | 0.019 | (0.253, 0.328) | 0.038 | 6.625 | 5620341 |
| 4 | Gujarat | 0.330 | 0.019 | (0.293, 0.367) | 0.037 | 5.764 | 5767531 |
| 5 | Haryana | 0.144 | 0.012 | (0.121, 0.167) | 0.023 | 8.181 | 1093000 |
| 6 | Himachal Pradesh | 0.051 | 0.007 | (0.037, 0.065) | 0.014 | 13.920 | 153495 |
| 7 | Jammu & Kashmir | 0.311 | 0.054 | (0.205, 0.417) | 0.106 | 17.428 | 1337066 |
| 8 | Karnataka | 0.458 | 0.013 | (0.433, 0.484) | 0.025 | 2.829 | 6628549 |
| 9 | Kerala | 0.092 | 0.010 | (0.073, 0.111) | 0.019 | 10.461 | 159045 |
| 10 | Madhya Pradesh | 0.341 | 0.008 | (0.325, 0.357) | 0.016 | 2.410 | 10359268 |
| 11 | Maharashtra | 0.372 | 0.014 | (0.345, 0.399) | 0.027 | 3.672 | 8196434 |
| 12 | Manipur | 0.176 | 0.017 | (0.142, 0.209) | 0.033 | 9.682 | 68370 |
| 13 | Mizoram | 0.070 | 0.009 | (0.053, 0.088) | 0.018 | 12.767 | 2856 |
| 14 | Nagaland | 0.200 | 0.029 | (0.144, 0.256) | 0.056 | 14.359 | 818708 |
| 15 | Odisha | 0.395 | 0.009 | (0.377, 0.413) | 0.018 | 2.346 | 5245755 |
| 16 | Punjab | 0.052 | 0.018 | (0.017, 0.087) | 0.035 | 34.542 | 360707 |
| 17 | Rajasthan | 0.368 | 0.031 | (0.308, 0.429) | 0.060 | 8.348 | 8779367 |
| 18 | Tamil Nadu | 0.244 | 0.023 | (0.200, 0.289) | 0.044 | 9.279 | 3050005 |
| 19 | Tripura | 0.113 | 0.016 | (0.081, 0.144) | 0.031 | 14.217 | 108742 |
| 20 | West Bengal | 0.309 | 0.018 | (0.274, 0.344) | 0.035 | 5.839 | 5989249 |

$\hat{P}_{i}$: Estimated value of DIVA positivity rate; $SE(\hat{P}_{i})$: Estimated value of standard error of $\hat{P}_{i}$; 95% CI of $\hat{P}_{i}$: 95% confidence interval of $\hat{P}_{i}$; $ME(\hat{P}_{i})$: Estimated value of error margin for $\hat{P}_{i}$; $CV(\hat{P}_{i})$: co-efficient of variation of $\hat{P}_{i}$; $\hat{Y}_{i}$: estimated number of DIVA positive animals

**Table S2**. State-wise estimated values of DIVA positive rates for the year 2009.

| **Sl. No.** | **STATE** | ${\hat{\boldsymbol{P}}}_{\boldsymbol{i}}$ | $\boldsymbol{SE(}{\hat{\boldsymbol{P}}}_{\boldsymbol{i}}\boldsymbol{)}$ | **95% CI of** ${\hat{\boldsymbol{P}}}_{\boldsymbol{i}}$ | $\boldsymbol{ME(}{\hat{\boldsymbol{P}}}_{\boldsymbol{i}}\boldsymbol{)}$ | $\boldsymbol{CV(}{\hat{\boldsymbol{P}}}_{\boldsymbol{i}}\boldsymbol{)}$ | ${\hat{\boldsymbol{Y}}}_{\boldsymbol{i}}$ |
| --- | --- | --- | --- | --- | --- | --- | --- |
| 1 | Andhra Pradesh | 0.214 | 0.010 | (0.194, 0.233) | 0.019 | 4.66 | 4865159 |
| 2 | Arunachal Pradesh | 0.299 | 0.017 | (0.266, 0.333) | 0.034 | 5.71 | 147174 |
| 3 | Assam | 0.331 | 0.022 | (0.288, 0.375) | 0.044 | 6.71 | 3516979 |
| 4 | Bihar | 0.267 | 0.007 | (0.253, 0.281) | 0.014 | 2.68 | 5198503 |
| 5 | Gujarat | 0.381 | 0.011 | (0.360, 0.403) | 0.021 | 2.85 | 6941621 |
| 6 | Haryana | 0.263 | 0.017 | (0.230, 0.297) | 0.033 | 6.44 | 2017080 |
| 7 | Himachal Pradesh | 0.051 | 0.007 | (0.037, 0.065) | 0.014 | 13.92 | 151798 |
| 8 | Jammu & Kashmir | 0.311 | 0.054 | (0.205, 0.417) | 0.106 | 17.43 | 1277658 |
| 9 | Karnataka | 0.484 | 0.011 | (0.463, 0.505) | 0.021 | 2.25 | 6822544 |
| 10 | Kerala | 0.152 | 0.010 | (0.133, 0.171) | 0.019 | 6.29 | 250779 |
| 11 | Madhya Pradesh | 0.297 | 0.007 | (0.282, 0.311) | 0.014 | 2.44 | 8821435 |
| 12 | Maharashtra | 0.298 | 0.009 | (0.281, 0.315) | 0.017 | 2.92 | 6482326 |
| 13 | Manipur | 0.160 | 0.012 | (0.137, 0.183) | 0.023 | 7.24 | 59858 |
| 14 | Mizoram | 0.070 | 0.009 | (0.053, 0.088) | 0.018 | 12.77 | 2838 |
| 15 | Nagaland | 0.230 | 0.017 | (0.196, 0.264) | 0.034 | 7.59 | 722282 |
| 16 | Odisha | 0.395 | 0.009 | (0.377 0.413) | 0.018 | 2.35 | 5154639 |
| 17 | Punjab | 0.125 | 0.007 | (0.110 0.139) | 0.014 | 5.85 | 889258 |
| 18 | Rajasthan | 0.563 | 0.023 | (0.517 0.608) | 0.045 | 4.11 | 13758290 |
| 19 | Tamil Nadu | 0.244 | 0.023 | (0.200 0.289) | 0.044 | 9.28 | 2873833 |
| 20 | Tripura | 0.113 | 0.016 | (0.081 0.144) | 0.031 | 14.22 | 108553 |
| 21 | West Bengal | 0.309 | 0.018 | (0.274 0.344) | 0.035 | 5.84 | 5813724 |

$\hat{P}_{i}$: Estimated value of DIVA positivity rate; $SE(\hat{P}_{i})$: Estimated value of standard error of $\hat{P}_{i}$; 95% CI of $\hat{P}_{i}$: 95% confidence interval of $\hat{P}_{i}$; $ME(\hat{P}_{i})$: Estimated value of error margin for $\hat{P}_{i}$; $CV(\hat{P}_{i})$: co-efficient of variation of $\hat{P}_{i}$; $\hat{Y}_{i}$: estimated number of DIVA positive animals in each state

**Table S3**. State-wise estimated values of DIVA positive rates for the year 2010.

| **Sl. No.** | **STATE** | ${\hat{\boldsymbol{P}}}_{\boldsymbol{i}}$ | $\boldsymbol{SE(}{\hat{\boldsymbol{P}}}_{\boldsymbol{i}}\boldsymbol{)}$ | **95% CI of** ${\hat{\boldsymbol{P}}}_{\boldsymbol{i}}$ | $\boldsymbol{ME(}{\hat{\boldsymbol{P}}}_{\boldsymbol{i}}\boldsymbol{)}$ | $\boldsymbol{CV(}{\hat{\boldsymbol{P}}}_{\boldsymbol{i}}\boldsymbol{)}$ | ${\hat{\boldsymbol{Y}}}_{\boldsymbol{i}}$ |
| --- | --- | --- | --- | --- | --- | --- | --- |
| 1 | Andhra Pradesh | 0.194 | 0.008 | (0.179, 0.209) | 0.015 | 3.98 | 4257049 |
| 2 | Arunachal Pradesh | 0.343 | 0.027 | (0.290, 0.395) | 0.052 | 7.81 | 166024 |
| 3 | Assam | 0.361 | 0.014 | (0.333, 0.388) | 0.027 | 3.87 | 3845208 |
| 4 | Bihar | 0.251 | 0.007 | (0.238, 0.265) | 0.014 | 2.76 | 4915968 |
| 5 | Gujarat | 0.193 | 0.008 | (0.178, 0.207) | 0.015 | 3.92 | 3645723 |
| 6 | Haryana | 0.119 | 0.007 | (0.105, 0.133) | 0.014 | 6.00 | 922991 |
| 7 | Himachal Pradesh | 0.097 | 0.011 | (0.077, 0.118) | 0.021 | 10.83 | 285735 |
| 8 | Jammu & Kashmir | 0.194 | 0.010 | (0.174, 0.214) | 0.020 | 5.26 | 761415 |
| 9 | Karnataka | 0.461 | 0.009 | (0.443, 0.480) | 0.018 | 2.04 | 6329333 |
| 10 | Kerala | 0.478 | 0.022 | (0.436, 0.520) | 0.042 | 4.50 | 753808 |
| 11 | Madhya Pradesh | 0.376 | 0.016 | (0.345, 0.407) | 0.031 | 4.19 | 10947870 |
| 12 | Maharashtra | 0.503 | 0.016 | (0.473, 0.534) | 0.031 | 3.13 | 10850029 |
| 13 | Manipur | 0.166 | 0.012 | (0.143, 0.189) | 0.023 | 7.13 | 59591 |
| 14 | Mizoram | 0.336 | 0.017 | (0.304, 0.369) | 0.032 | 4.92 | 13533 |
| 15 | Nagaland | 0.700 | 0.153 | (0.401, 0.999) | 0.299 | 21.82 | 1526431 |
| 16 | Odisha | 0.104 | 0.007 | (0.091, 0.117) | 0.013 | 6.35 | 1328737 |
| 17 | Punjab | 0.122 | 0.007 | (0.108, 0.136) | 0.014 | 6.00 | 889144 |
| 18 | Rajasthan | 0.530 | 0.023 | (0.486, 0.575) | 0.044 | 4.25 | 13296748 |
| 19 | Tamil Nadu | 0.197 | 0.011 | (0.176, 0.218) | 0.021 | 5.41 | 2175419 |
| 20 | Tripura | 0.114 | 0.013 | (0.089, 0.138) | 0.025 | 11.02 | 109495 |
| 21 | Uttar Pradesh | 0.280 | 0.013 | (0.255, 0.306) | 0.026 | 4.68 | 13235011 |
| 22 | Uttarakhand | 0.148 | 0.016 | (0.116, 0.179) | 0.032 | 10.89 | 469892 |
| 23 | West Bengal | 0.265 | 0.013 | (0.239, 0.291) | 0.026 | 5.05 | 4834736 |

$\hat{P}_{i}$: Estimated value of DIVA positivity rate; $SE(\hat{P}_{i})$: Estimated value of standard error of $\hat{P}_{i}$; 95% CI of $\hat{P}_{i}$: 95% confidence interval of $\hat{P}_{i}$; $ME(\hat{P}_{i})$: Estimated value of error margin for $\hat{P}_{i}$; $CV(\hat{P}_{i})$: co-efficient of variation of $\hat{P}_{i}$; $\hat{Y}_{i}$: estimated number of DIVA positive animals

**Table S4**. State-wise estimated values of DIVA positive rates for the year 2011.

| **Sl. No.** | **STATE** | ${\hat{\boldsymbol{P}}}_{\boldsymbol{i}}$ | $\boldsymbol{SE(}{\hat{\boldsymbol{P}}}_{\boldsymbol{i}}\boldsymbol{)}$ | **95% CI of** ${\hat{\boldsymbol{P}}}_{\boldsymbol{i}}$ | $\boldsymbol{ME(}{\hat{\boldsymbol{P}}}_{\boldsymbol{i}}\boldsymbol{)}$ | $\boldsymbol{CV(}{\hat{\boldsymbol{P}}}_{\boldsymbol{i}}\boldsymbol{)}$ | ${\hat{\boldsymbol{Y}}}_{\boldsymbol{i}}$ |
| --- | --- | --- | --- | --- | --- | --- | --- |
| 1 | Andhra Pradesh | 0.244 | 0.009 | (0.226, 0.262) | 0.018 | 3.757 | 5134389 |
| 2 | Arunachal Pradesh | 0.348 | 0.024 | (0.302, 0.394) | 0.046 | 6.781 | 166009 |
| 3 | Assam | 0.319 | 0.011 | (0.298, 0.340) | 0.021 | 3.353 | 3413533 |
| 4 | Bihar | 0.275 | 0.009 | (0.257, 0.293) | 0.018 | 3.388 | 5410141 |
| 5 | Gujarat | 0.403 | 0.010 | (0.382, 0.423) | 0.020 | 2.551 | 7909948 |
| 6 | Haryana | 0.083 | 0.004 | (0.075, 0.092) | 0.009 | 5.335 | 650302 |
| 7 | Himachal Pradesh | 0.065 | 0.007 | (0.051, 0.079) | 0.014 | 11.096 | 187632 |
| 8 | Jammu & Kashmir | 0.141 | 0.012 | (0.117, 0.165) | 0.024 | 8.647 | 525456 |
| 9 | Karnataka | 0.581 | 0.009 | (0.564, 0.599) | 0.017 | 1.530 | 7762558 |
| 10 | Kerala | 0.228 | 0.013 | (0.202, 0.254) | 0.026 | 5.820 | 342984 |
| 11 | Madhya Pradesh | 0.276 | 0.008 | (0.260, 0.292) | 0.016 | 2.888 | 7852055 |
| 12 | Maharashtra | 0.444 | 0.012 | (0.420, 0.468) | 0.024 | 2.782 | 9459856 |
| 13 | Manipur | 0.207 | 0.013 | (0.180, 0.233) | 0.026 | 6.526 | 71294 |
| 14 | Meghalaya | 0.310 | 0.027 | (0.258, 0.362) | 0.052 | 8.627 | 284099 |
| 15 | Mizoram | 0.273 | 0.015 | (0.244, 0.302) | 0.029 | 5.447 | 10908 |
| 16 | Nagaland | 0.125 | 0.005 | (0.115, 0.136) | 0.010 | 4.176 | 153403 |
| 17 | Odisha | 0.101 | 0.006 | (0.090, 0.112) | 0.011 | 5.539 | 1270818 |
| 18 | Rajasthan | 0.368 | 0.011 | (0.346, 0.389) | 0.021 | 2.958 | 9440111 |
| 19 | Tamil Nadu | 0.288 | 0.008 | (0.271, 0.304) | 0.016 | 2.873 | 2967333 |
| 20 | Tripura | 0.109 | 0.015 | (0.080, 0.138) | 0.029 | 13.494 | 105140 |
| 21 | Uttar Pradesh | 0.133 | 0.025 | (0.083, 0.182) | 0.050 | 19.064 | 6455459 |
| 22 | Uttarakhand | 0.293 | 0.021 | (0.251, 0.335) | 0.042 | 7.324 | 905245 |
| 23 | West Bengal | 0.320 | 0.017 | (0.286, 0.354) | 0.034 | 5.428 | 5664368 |

$\hat{P}_{i}$: Estimated value of DIVA positivity rate; $SE(\hat{P}_{i})$: Estimated value of standard error of $\hat{P}_{i}$; 95% CI of $\hat{P}_{i}$: 95% confidence interval of $\hat{P}_{i}$; $ME(\hat{P}_{i})$: Estimated value of error margin for $\hat{P}_{i}$; $CV(\hat{P}_{i})$: co-efficient of variation of $\hat{P}_{i}$; $\hat{Y}_{i}$: estimated number of DIVA positive animals in each state

**Table S5**. State-wise estimated values of DIVA positive rates for the year 2012.

| **Sl. No.** | **STATE** | ${\hat{\boldsymbol{P}}}_{\boldsymbol{i}}$ | $\boldsymbol{SE(}{\hat{\boldsymbol{P}}}_{\boldsymbol{i}}\boldsymbol{)}$ | **95% CI of** ${\hat{\boldsymbol{P}}}_{\boldsymbol{i}}$ | $\boldsymbol{ME(}{\hat{\boldsymbol{P}}}_{\boldsymbol{i}}\boldsymbol{)}$ | $\boldsymbol{CV(}{\hat{\boldsymbol{P}}}_{\boldsymbol{i}}\boldsymbol{)}$ | ${\hat{\boldsymbol{Y}}}_{\boldsymbol{i}}$ |
| --- | --- | --- | --- | --- | --- | --- | --- |
| 1 | Andhra Pradesh | 0.232 | 0.010 | (0.211, 0.252) | 0.020 | 4.486 | 4680943 |
| 2 | Arunachal Pradesh | 0.344 | 0.017 | (0.311, 0.378) | 0.033 | 4.918 | 161749 |
| 3 | Assam | 0.245 | 0.012 | (0.221, 0.269) | 0.024 | 5.069 | 2632003 |
| 4 | Bihar | 0.319 | 0.010 | (0.299, 0.339) | 0.020 | 3.209 | 6307400 |
| 5 | Gujarat | 0.411 | 0.010 | (0.391, 0.431) | 0.020 | 2.475 | 8370562 |
| 6 | Haryana | 0.070 | 0.004 | (0.063, 0.078) | 0.008 | 5.613 | 554419 |
| 7 | Himachal Pradesh | 0.089 | 0.015 | (0.061, 0.118) | 0.029 | 16.385 | 256367 |
| 8 | Jammu &Kashmir | 0.168 | 0.012 | (0.144, 0.192) | 0.024 | 7.331 | 593381 |
| 9 | Jharkhand | 0.353 | 0.016 | (0.322, 0.385) | 0.031 | 4.483 | 3504896 |
| 10 | Karnataka | 0.176 | 0.007 | (0.162, 0.191) | 0.014 | 4.125 | 2290708 |
| 11 | Kerala | 0.299 | 0.013 | (0.274, 0.324) | 0.025 | 4.311 | 428133 |
| 12 | Madhya Pradesh | 0.286 | 0.009 | (0.268, 0.303) | 0.017 | 3.064 | 7935632 |
| 13 | Maharashtra | 0.218 | 0.011 | (0.197, 0.240) | 0.021 | 5.007 | 4598967 |
| 14 | Manipur | 0.244 | 0.014 | (0.217, 0.271) | 0.027 | 5.691 | 80565 |
| 15 | Meghalaya | 0.230 | 0.042 | (0.147, 0.313) | 0.083 | 18.388 | 211154 |
| 16 | Mizoram | 0.158 | 0.013 | (0.132, 0.183) | 0.025 | 8.099 | 6260 |
| 17 | Nagaland | 0.405 | 0.015 | (0.376, 0.434) | 0.029 | 3.614 | 108415 |
| 18 | Odisha | 0.342 | 0.009 | (0.323, 0.360) | 0.018 | 2.763 | 4216842 |
| 19 | Punjab | 0.114 | 0.008 | (0.099, 0.129) | 0.015 | 6.759 | 865862 |
| 20 | Rajasthan | 0.400 | 0.009 | (0.383, 0.417) | 0.017 | 2.174 | 10525199 |
| 21 | Tamil Nadu | 0.462 | 0.009 | (0.445, 0.480) | 0.018 | 1.937 | 4435123 |
| 22 | Uttar Pradesh | 0.196 | 0.007 | (0.182, 0.211) | 0.014 | 3.745 | 9857565 |
| 23 | West Bengal | 0.246 | 0.013 | (0.220, 0.272) | 0.026 | 5.336 | 4210380 |

$\hat{P}_{i}$: Estimated value of DIVA positivity rate; $SE(\hat{P}_{i})$: Estimated value of standard error of $\hat{P}_{i}$; 95% CI of $\hat{P}_{i}$: 95% confidence interval of $\hat{P}_{i}$; $ME(\hat{P}_{i})$: Estimated value of error margin for $\hat{P}_{i}$; $CV(\hat{P}_{i})$: co-efficient of variation of $\hat{P}_{i}$; $\hat{Y}_{i}$: estimated number of DIVA positive animals in each state

**Table S6**. State-wise estimated values of DIVA positive rates for the year 2013.

| **Sl. No.** | **STATE** | ${\hat{\boldsymbol{P}}}_{\boldsymbol{i}}$ | $\boldsymbol{SE(}{\hat{\boldsymbol{P}}}_{\boldsymbol{i}}\boldsymbol{)}$ | **95% CI of** ${\hat{\boldsymbol{P}}}_{\boldsymbol{i}}$ | $\boldsymbol{ME(}{\hat{\boldsymbol{P}}}_{\boldsymbol{i}}\boldsymbol{)}$ | $\boldsymbol{CV(}{\hat{\boldsymbol{P}}}_{\boldsymbol{i}}\boldsymbol{)}$ | ${\hat{\boldsymbol{Y}}}_{\boldsymbol{i}}$ |
| --- | --- | --- | --- | --- | --- | --- | --- |
| 1 | Andhra Pradesh | 0.428 | 0.011 | (0.407, 0.449) | 0.021 | 2.472 | 8599726 |
| 2 | Arunachal Pradesh | 0.288 | 0.015 | (0.259, 0.318) | 0.030 | 5.260 | 130374 |
| 3 | Assam | 0.177 | 0.007 | (0.162, 0.191) | 0.015 | 4.236 | 1911361 |
| 4 | Bihar | 0.364 | 0.010 | (0.344, 0.384) | 0.020 | 2.785 | 7385064 |
| 5 | Gujarat | 0.605 | 0.010 | (0.586, 0.624) | 0.019 | 1.617 | 12302846 |
| 6 | Haryana | 0.021 | 0.002 | (0.016, 0.025) | 0.004 | 10.608 | 158782 |
| 7 | Himachal Pradesh | 0.128 | 0.010 | (0.109, 0.147) | 0.019 | 7.525 | 360548 |
| 8 | Jammu & Kashmir | 0.186 | 0.010 | (0.166, 0.207) | 0.020 | 5.584 | 650809 |
| 9 | Jharkhand | 0.338 | 0.020 | (0.299, 0.377) | 0.039 | 5.939 | 3481261 |
| 10 | Karnataka | 0.211 | 0.007 | (0.196, 0.226) | 0.015 | 3.536 | 2693602 |
| 11 | Kerala | 0.444 | 0.014 | (0.416, 0.471) | 0.027 | 3.155 | 635617 |
| 12 | Madhya Pradesh | 0.293 | 0.007 | (0.280, 0.306) | 0.013 | 2.300 | 8198738 |
| 13 | Maharashtra | 0.484 | 0.009 | (0.466, 0.502) | 0.018 | 1.897 | 10102292 |
| 14 | Manipur | 0.283 | 0.015 | (0.254, 0.313) | 0.029 | 5.297 | 90747 |
| 15 | Mizoram | 0.075 | 0.011 | (0.053, 0.096) | 0.022 | 14.936 | 3049 |
| 16 | Nagaland | 0.323 | 0.008 | (0.306, 0.339) | 0.017 | 2.617 | 78390 |
| 17 | Odisha | 0.415 | 0.009 | (0.398, 0.432) | 0.017 | 2.058 | 5009121 |
| 18 | Punjab | 0.121 | 0.007 | (0.106, 0.136) | 0.015 | 6.183 | 900495 |
| 19 | Rajasthan | 0.374 | 0.006 | (0.361, 0.387) | 0.013 | 1.729 | 9246948 |
| 20 | Tamil Nadu | 0.200 | 0.007 | (0.185, 0.214) | 0.014 | 3.625 | 1928385 |
| 21 | Tripura | 0.083 | 0.008 | (0.067, 0.099) | 0.016 | 9.824 | 77020 |
| 22 | Uttar Pradesh | 0.564 | 0.016 | (0.534, 0.594) | 0.030 | 2.750 | 28453636 |
| 23 | Uttarakhand | 0.320 | 0.022 | (0.277, 0.363) | 0.043 | 6.903 | 945171 |
| 24 | West Bengal | 0.325 | 0.025 | (0.277, 0.373) | 0.048 | 7.606 | 5681861 |

$\hat{P}_{i}$: Estimated value of DIVA positivity rate; $SE(\hat{P}_{i})$: Estimated value of standard error of $\hat{P}_{i}$; 95% CI of $\hat{P}_{i}$: 95% confidence interval of $\hat{P}_{i}$; $ME(\hat{P}_{i})$: Estimated value of error margin for $\hat{P}_{i}$; $CV(\hat{P}_{i})$: co-efficient of variation of $\hat{P}_{i}$; $\hat{Y}_{i}$: estimated number of DIVA positive animals in each state

**Table S7**. State-wise estimated values of DIVA positive rates for the year 2014.

| **Sl. No.** | **STATE** | ${\hat{\boldsymbol{P}}}_{\boldsymbol{i}}$ | $\boldsymbol{SE(}{\hat{\boldsymbol{P}}}_{\boldsymbol{i}}\boldsymbol{)}$ | **95% CI of** ${\hat{\boldsymbol{P}}}_{\boldsymbol{i}}$ | $\boldsymbol{ME(}{\hat{\boldsymbol{P}}}_{\boldsymbol{i}}\boldsymbol{)}$ | $\boldsymbol{CV(}{\hat{\boldsymbol{P}}}_{\boldsymbol{i}}\boldsymbol{)}$ | ${\hat{\boldsymbol{Y}}}_{\boldsymbol{i}}$ |
| --- | --- | --- | --- | --- | --- | --- | --- |
| 1 | Andhra Pradesh | 0.305 | 0.007 | (0.291,0.319) | 0.014 | 2.276 | 6084773 |
| 2 | Arunachal Pradesh | 0.141 | 0.038 | (0.067,0.216) | 0.074 | 26.908 | 61308 |
| 3 | Assam | 0.194 | 0.008 | (0.177,0.210) | 0.016 | 4.296 | 2111630 |
| 4 | Bihar | 0.348 | 0.021 | (0.308,0.388) | 0.040 | 5.894 | 7223046 |
| 5 | Chhattisgarh | 0.637 | 0.031 | (0.577,0.697) | 0.060 | 4.802 | 7130441 |
| 6 | Gujarat | 0.176 | 0.005 | (0.165,0.186) | 0.011 | 3.064 | 3567224 |
| 7 | Haryana | 0.055 | 0.004 | (0.048,0.062) | 0.007 | 6.707 | 409926 |
| 8 | Himachal Pradesh | 0.134 | 0.007 | (0.121,0.148) | 0.014 | 5.184 | 369448 |
| 9 | Jammu & Kashmir | 0.304 | 0.013 | (0.279,0.329) | 0.025 | 4.264 | 1048548 |
| 10 | Karnataka | 0.332 | 0.006 | (0.320,0.344) | 0.012 | 1.830 | 4169017 |
| 11 | Kerala | 0.069 | 0.006 | (0.058,0.080) | 0.011 | 8.272 | 98481 |
| 12 | Madhya Pradesh | 0.257 | 0.005 | (0.247,0.266) | 0.009 | 1.853 | 7226817 |
| 13 | Maharashtra | 0.283 | 0.007 | (0.269,0.296) | 0.014 | 2.453 | 5838453 |
| 14 | Manipur | 0.130 | 0.008 | (0.115,0.145) | 0.015 | 6.081 | 40346 |
| 15 | Mizoram | 0.098 | 0.007 | (0.084,0.111) | 0.014 | 7.202 | 4107 |
| 16 | Nagaland | 0.337 | 0.016 | (0.306,0.368) | 0.031 | 4.702 | 73504 |
| 17 | Odisha | 0.332 | 0.008 | (0.315,0.348) | 0.016 | 2.514 | 3905865 |
| 18 | Punjab | 0.097 | 0.007 | (0.084,0.111) | 0.013 | 6.986 | 709844 |
| 19 | Rajasthan | 0.266 | 0.006 | (0.255,0.277) | 0.011 | 2.120 | 6155826 |
| 20 | Tamil Nadu | 0.243 | 0.005 | (0.232,0.253) | 0.011 | 2.208 | 2358871 |
| 21 | Tripura | 0.043 | 0.005 | (0.033,0.052) | 0.009 | 11.290 | 38402 |
| 22 | Uttar Pradesh | 0.459 | 0.011 | (0.437,0.481) | 0.022 | 2.454 | 23272179 |
| 23 | Uttarakhand | 0.412 | 0.016 | (0.381,0.443) | 0.031 | 3.797 | 1201394 |
| 24 | West Bengal | 0.177 | 0.012 | (0.154,0.201) | 0.023 | 6.690 | 3164814 |

$\hat{P}_{i}$: Estimated value of DIVA positivity rate; $SE(\hat{P}_{i})$: Estimated value of standard error of $\hat{P}_{i}$; 95% CI of $\hat{P}_{i}$: 95% confidence interval of $\hat{P}_{i}$; $ME(\hat{P}_{i})$: Estimated value of error margin for $\hat{P}_{i}$; $CV(\hat{P}_{i})$: co-efficient of variation of $\hat{P}_{i}$; $\hat{Y}_{i}$: estimated number of DIVA positive animals in each state

**Table S8**. State-wise estimated values of DIVA positive rates for the year 2015.

| **Sl. No.** | **STATE** | ${\hat{\boldsymbol{P}}}_{\boldsymbol{i}}$ | $\boldsymbol{SE(}{\hat{\boldsymbol{P}}}_{\boldsymbol{i}}\boldsymbol{)}$ | **95% CI of** ${\hat{\boldsymbol{P}}}_{\boldsymbol{i}}$ | $\boldsymbol{ME(}{\hat{\boldsymbol{P}}}_{\boldsymbol{i}}\boldsymbol{)}$ | $\boldsymbol{CV(}{\hat{\boldsymbol{P}}}_{\boldsymbol{i}}\boldsymbol{)}$ | ${\hat{\boldsymbol{Y}}}_{\boldsymbol{i}}$ |
| --- | --- | --- | --- | --- | --- | --- | --- |
| 1 | Assam | 0.167 | 0.007 | (0.154, 0.180) | 0.013 | 4.102 | 1835574 |
| 2 | Gujarat | 0.396 | 0.008 | (0.382, 0.411) | 0.015 | 1.909 | 8041987 |
| 3 | Haryana | 0.090 | 0.006 | (0.078, 0.102) | 0.012 | 6.940 | 648821 |
| 4 | Himachal Pradesh | 0.109 | 0.006 | (0.096,0.121) | 0.012 | 5.842 | 293390 |
| 5 | Jammu & Kashmir | 0.274 | 0.011 | (0.252,0.296) | 0.022 | 4.072 | 932293 |
| 6 | Karnataka | 0.341 | 0.006 | (0.330,0.353) | 0.012 | 1.792 | 4210528 |
| 7 | Madhya Pradesh | 0.203 | 0.005 | (0.194,0.212) | 0.009 | 2.237 | 5762339 |
| 8 | Maharashtra | 0.204 | 0.011 | (0.183,0.225) | 0.021 | 5.306 | 4165173 |
| 9 | Mizoram | 0.069 | 0.009 | (0.051,0.086) | 0.017 | 12.899 | 2970 |
| 10 | Odisha | 0.193 | 0.006 | (0.181,0.205) | 0.012 | 3.192 | 2219210 |
| 11 | Punjab | 0.091 | 0.005 | (0.082,0.100) | 0.009 | 5.170 | 648553 |
| 12 | Rajasthan | 0.381 | 0.009 | (0.363,0.399) | 0.018 | 2.453 | 8226831 |
| 13 | Tamil Nadu | 0.272 | 0.006 | (0.261,0.283) | 0.011 | 2.043 | 2664699 |
| 14 | Telangana | 0.338 | 0.011 | (0.316,0.360) | 0.022 | 3.301 | 2857210 |
| 15 | Tripura | 0.038 | 0.006 | (0.027,0.049) | 0.011 | 14.958 | 32574 |
| 16 | Uttar Pradesh | 0.189 | 0.004 | (0.180,0.197) | 0.009 | 2.312 | 9612803 |
| 17 | Uttarakhand | 0.328 | 0.015 | (0.299,0.358) | 0.030 | 4.626 | 944564 |
| 18 | West Bengal | 0.173 | 0.006 | (0.161,0.185) | 0.012 | 3.447 | 3152970 |

$\hat{P}_{i}$: Estimated value of DIVA positivity rate; $SE(\hat{P}_{i})$: Estimated value of standard error of $\hat{P}_{i}$; 95% CI of $\hat{P}_{i}$: 95% confidence interval of $\hat{P}_{i}$; $ME(\hat{P}_{i})$: Estimated value of error margin for $\hat{P}_{i}$; $CV(\hat{P}_{i})$: co-efficient of variation of $\hat{P}_{i}$; $\hat{Y}_{i}$: estimated number of DIVA positive animals in each state

**Table S9**. State-wise estimated values of DIVA positive rates for the year 2016.

| **Sl. No.** | **STATE** | ${\hat{\boldsymbol{P}}}_{\boldsymbol{i}}$ | $\boldsymbol{SE(}{\hat{\boldsymbol{P}}}_{\boldsymbol{i}}\boldsymbol{)}$ | **95% CI of** ${\hat{\boldsymbol{P}}}_{\boldsymbol{i}}$ | $\boldsymbol{ME(}{\hat{\boldsymbol{P}}}_{\boldsymbol{i}}\boldsymbol{)}$ | $\boldsymbol{CV(}{\hat{\boldsymbol{P}}}_{\boldsymbol{i}}\boldsymbol{)}$ | ${\hat{\boldsymbol{Y}}}_{\boldsymbol{i}}$ |
| --- | --- | --- | --- | --- | --- | --- | --- |
| 1 | Andhra Pradesh | 0.225 | 0.008 | (0.209,0.241) | 0.016 | 3.636 | 2529395 |
| 2 | Assam | 0.371 | 0.016 | (0.339,0.403) | 0.032 | 4.414 | 4113207 |
| 3 | Gujarat | 0.209 | 0.008 | (0.194,0.224) | 0.015 | 3.682 | 4225542 |
| 4 | Haryana | 0.576 | 0.034 | (0.510,0.642) | 0.066 | 5.837 | 4021322 |
| 5 | Himachal Pradesh | 0.082 | 0.007 | (0.069,0.095) | 0.013 | 8.371 | 216586 |
| 6 | Karnataka | 0.308 | 0.006 | (0.296,0.320) | 0.012 | 1.940 | 3728723 |
| 7 | Kerala | 0.175 | 0.008 | (0.161,0.190) | 0.015 | 4.316 | 252238 |
| 8 | Madhya Pradesh | 0.203 | 0.004 | (0.195,0.211) | 0.008 | 2.035 | 5784720 |
| 9 | Maharashtra | 0.259 | 0.007 | (0.246,0.272) | 0.013 | 2.532 | 5235616 |
| 10 | Manipur | 0.126 | 0.008 | (0.111,0.141) | 0.015 | 6.187 | 36634 |
| 11 | Mizoram | 0.142 | 0.013 | (0.117,0.167) | 0.025 | 8.842 | 6303 |
| 12 | Odisha | 0.336 | 0.011 | (0.314,0.358) | 0.022 | 3.390 | 3765944 |
| 13 | Punjab | 0.099 | 0.007 | (0.086,0.112) | 0.013 | 6.713 | 692047 |
| 14 | Rajasthan | 0.357 | 0.010 | (0.338,0.377) | 0.019 | 2.773 | 7150681 |
| 15 | Tamil Nadu | 0.277 | 0.006 | (0.266,0.289) | 0.011 | 2.084 | 2731069 |
| 16 | Telangana | 0.090 | 0.007 | (0.077,0.103) | 0.013 | 7.496 | 761296 |
| 17 | Tripura | 0.031 | 0.006 | (0.020,0.042) | 0.011 | 18.602 | 26060 |
| 18 | Uttar Pradesh | 0.158 | 0.004 | (0.150,0.165) | 0.008 | 2.429 | 8088729 |
| 19 | Uttarakhand | 0.140 | 0.014 | (0.112,0.168) | 0.028 | 10.124 | 397768 |
| 20 | West Bengal | 0.337 | 0.009 | (0.318,0.355) | 0.019 | 2.812 | 6260766 |

$\hat{P}_{i}$: Estimated value of DIVA positivity rate; $SE(\hat{P}_{i})$: Estimated value of standard error of $\hat{P}_{i}$; 95% CI of $\hat{P}_{i}$: 95% confidence interval of $\hat{P}_{i}$; $ME(\hat{P}_{i})$: Estimated value of error margin for $\hat{P}_{i}$; $CV(\hat{P}_{i})$: co-efficient of variation of $\hat{P}_{i}$; $\hat{Y}_{i}$: estimated number of DIVA positive animals in each state

**Table S10**. State-wise estimated values of DIVA positive rates for the year 2017.

| **Sl. No.** | **STATE** | ${\hat{\boldsymbol{P}}}_{\boldsymbol{i}}$ | $\boldsymbol{SE(}{\hat{\boldsymbol{P}}}_{\boldsymbol{i}}\boldsymbol{)}$ | **95% CI of** ${\hat{\boldsymbol{P}}}_{\boldsymbol{i}}$ | $\boldsymbol{ME(}{\hat{\boldsymbol{P}}}_{\boldsymbol{i}}\boldsymbol{)}$ | $\boldsymbol{CV(}{\hat{\boldsymbol{P}}}_{\boldsymbol{i}}\boldsymbol{)}$ | ${\hat{\boldsymbol{Y}}}_{\boldsymbol{i}}$ |
| --- | --- | --- | --- | --- | --- | --- | --- |
| 1 | Assam | 0.190 | 0.009 | (0.172,0.209) | 0.019 | 4.970 | 2121345 |
| 2 | Haryana | 0.323 | 0.041 | (0.242,0.404) | 0.081 | 12.744 | 2181710 |
| 3 | Himachal Pradesh | 0.267 | 0.009 | (0.248,0.285) | 0.018 | 3.528 | 689266 |
| 4 | Jammu & Kashmir | 0.184 | 0.009 | (0.166,0.203) | 0.018 | 5.089 | 611184 |
| 5 | Jharkhand | 0.562 | 0.021 | (0.521,0.603) | 0.041 | 3.749 | 6641454 |
| 6 | Madhya Pradesh | 0.180 | 0.004 | (0.172,0.187) | 0.007 | 2.122 | 5155169 |
| 7 | Maharashtra | 0.059 | 0.004 | (0.051,0.068) | 0.009 | 7.505 | 1191048 |
| 8 | Manipur | 0.130 | 0.008 | (0.115,0.145) | 0.015 | 6.080 | 36473 |
| 9 | Mizoram | 0.099 | 0.010 | (0.078,0.119) | 0.021 | 10.593 | 4494 |
| 10 | Odisha | 0.621 | 0.009 | (0.603,0.639) | 0.018 | 1.503 | 6788445 |
| 11 | Punjab | 0.095 | 0.006 | (0.083,0.108) | 0.013 | 6.812 | 652979 |
| 12 | Rajasthan | 0.286 | 0.008 | (0.271,0.301) | 0.015 | 2.669 | 5270377 |
| 13 | Tamil Nadu | 0.253 | 0.005 | (0.243,0.264) | 0.011 | 2.145 | 2511791 |
| 14 | Telangana | 0.016 | 0.004 | (0.007,0.024) | 0.008 | 26.531 | 131582 |
| 15 | Uttar Pradesh | 0.099 | 0.006 | (0.088,0.111) | 0.012 | 6.016 | 5109629 |
| 16 | Uttarakhand | 0.070 | 0.008 | (0.054,0.085) | 0.015 | 11.141 | 194605 |
| 17 | West Bengal | 0.309 | 0.020 | (0.269,0.348) | 0.040 | 6.539 | 5852605 |

$\hat{P}_{i}$: Estimated value of DIVA positivity rate; $SE(\hat{P}_{i})$: Estimated value of standard error of $\hat{P}_{i}$; 95% CI of $\hat{P}_{i}$: 95% confidence interval of $\hat{P}_{i}$; $ME(\hat{P}_{i})$: Estimated value of error margin for $\hat{P}_{i}$; $CV(\hat{P}_{i})$: co-efficient of variation of $\hat{P}_{i}$; $\hat{Y}_{i}$: estimated number of DIVA positive animals in each state

**Table S11**. State-wise estimated values of DIVA positive rates for the year 2018.

| **Sl. No.** | **STATE** | ${\hat{\boldsymbol{P}}}_{\boldsymbol{i}}$ | $\boldsymbol{SE(}{\hat{\boldsymbol{P}}}_{\boldsymbol{i}}\boldsymbol{)}$ | **95% CI of** ${\hat{\boldsymbol{P}}}_{\boldsymbol{i}}$ | $\boldsymbol{ME(}{\hat{\boldsymbol{P}}}_{\boldsymbol{i}}\boldsymbol{)}$ | $\boldsymbol{CV(}{\hat{\boldsymbol{P}}}_{\boldsymbol{i}}\boldsymbol{)}$ | ${\hat{\boldsymbol{Y}}}_{\boldsymbol{i}}$ |
| --- | --- | --- | --- | --- | --- | --- | --- |
| 1 | Andhra Pradesh | 0.017 | 0.003 | (0.012,0.021) | 0.005 | 15.125 | 321057 |
| 2 | Assam | 0.204 | 0.015 | (0.175,0.233) | 0.029 | 7.313 | 2295609 |
| 3 | Gujarat | 0.416 | 0.007 | (0.403,0.430) | 0.013 | 1.643 | 8408142 |
| 4 | Haryana | 0.081 | 0.007 | (0.068,0.094) | 0.013 | 8.161 | 529661 |
| 5 | Himachal Pradesh | 0.199 | 0.022 | (0.157,0.242) | 0.042 | 10.786 | 504616 |
| 6 | Jammu & Kashmir | 0.246 | 0.010 | (0.226,0.265) | 0.020 | 4.131 | 803939 |
| 7 | Karnataka | 0.230 | 0.005 | (0.219,0.241) | 0.011 | 2.366 | 2684372 |
| 8 | Kerala | 0.053 | 0.006 | (0.041,0.064) | 0.011 | 10.949 | 75930 |
| 9 | Madhya Pradesh | 0.162 | 0.004 | (0.155,0.170) | 0.007 | 2.360 | 4680045 |
| 10 | Maharashtra | 0.037 | 0.003 | (0.030,0.044) | 0.007 | 9.358 | 732143 |
| 11 | Meghalaya | 0.302 | 0.050 | (0.205,0.400) | 0.098 | 16.476 | 277870 |
| 12 | Odisha | 0.457 | 0.016 | (0.426,0.487) | 0.031 | 3.427 | 4861000 |
| 13 | Punjab | 0.102 | 0.005 | (0.092,0.112) | 0.010 | 4.823 | 682326 |
| 14 | Tamil Nadu | 0.300 | 0.006 | (0.289,0.311) | 0.011 | 1.910 | 2990693 |
| 15 | Telangana | 0.010 | 0.002 | (0.005,0.015) | 0.005 | 24.136 | 84838 |
| 16 | Uttar Pradesh | 0.094 | 0.004 | (0.085,0.103) | 0.009 | 4.730 | 4859208 |
| 17 | West Bengal | 0.509 | 0.011 | (0.487,0.531) | 0.022 | 2.200 | 9843467 |

$\hat{P}_{i}$: Estimated value of DIVA positivity rate; $SE(\hat{P}_{i})$: Estimated value of standard error of $\hat{P}_{i}$; 95% CI of $\hat{P}_{i}$: 95% confidence interval of $\hat{P}_{i}$; $ME(\hat{P}_{i})$: Estimated value of error margin for $\hat{P}_{i}$; $CV(\hat{P}_{i})$: co-efficient of variation of $\hat{P}_{i}$; $\hat{Y}_{i}$: estimated number of DIVA positive animals in each state

**Table S12**. State-wise estimated values of DIVA positive rates for the year 2019.

| **Sl. No.** | **STATE** | ${\hat{\boldsymbol{P}}}_{\boldsymbol{i}}$ | $\boldsymbol{SE(}{\hat{\boldsymbol{P}}}_{\boldsymbol{i}}\boldsymbol{)}$ | **95% CI of** ${\hat{\boldsymbol{P}}}_{\boldsymbol{i}}$ | $\boldsymbol{ME(}{\hat{\boldsymbol{P}}}_{\boldsymbol{i}}\boldsymbol{)}$ | $\boldsymbol{CV(}{\hat{\boldsymbol{P}}}_{\boldsymbol{i}}\boldsymbol{)}$ | ${\hat{\boldsymbol{Y}}}_{\boldsymbol{i}}$ |
| --- | --- | --- | --- | --- | --- | --- | --- |
| 1 | Assam | 0.093 | 0.013 | (0.119,0.068) | 0.025 | 13.90 | 1058757 |
| 2 | Gujarat | 0.376 | 0.007 | (0.389,0.363) | 0.013 | 1.80 | 7581025 |
| 3 | Haryana | 0.073 | 0.005 | (0.083,0.063) | 0.010 | 7.20 | 460045 |
| 4 | Himachal Pradesh | 0.133 | 0.013 | (0.158,0.108) | 0.025 | 9.51 | 329944.3 |
| 5 | Jammu & Kashmir | 0.162 | 0.009 | (0.180,0.144) | 0.018 | 5.66 | 522393.9 |
| 6 | Madhya Pradesh | 0.146 | 0.004 | (0.154,0.137) | 0.009 | 3.04 | 4234945 |
| 7 | Maharashtra | 0.268 | 0.008 | (0.284,0.252) | 0.016 | 3.02 | 5253144 |
| 8 | Odisha | 0.275 | 0.018 | (0.310,0.239) | 0.036 | 6.64 | 2844889 |
| 9 | Tamil Nadu | 0.222 | 0.005 | (0.232,0.212) | 0.010 | 2.34 | 2227060 |
| 10 | Telangana | 0.034 | 0.004 | (0.042,0.026) | 0.008 | 12.59 | 286660.9 |
| 11 | West Bengal | 0.316 | 0.012 | (0.341,0.292) | 0.024 | 3.91 | 6234855 |

$\hat{P}_{i}$: Estimated value of DIVA positivity rate; $SE(\hat{P}_{i})$: Estimated value of standard error of $\hat{P}_{i}$; 95% CI of $\hat{P}_{i}$: 95% confidence interval of $\hat{P}_{i}$; $ME(\hat{P}_{i})$: Estimated value of error margin for $\hat{P}_{i}$; $CV(\hat{P}_{i})$: co-efficient of variation of $\hat{P}_{i}$; $\hat{Y}_{i}$: estimated number of DIVA positive animals in each state

**Table S13**. State-wise estimated values of DIVA positive rates for the year 2020.

| **Sl. No.** | **STATE** | ${\hat{\boldsymbol{P}}}_{\boldsymbol{i}}$ | $\boldsymbol{SE(}{\hat{\boldsymbol{P}}}_{\boldsymbol{i}}\boldsymbol{)}$ | **95% CI of** ${\hat{\boldsymbol{P}}}_{\boldsymbol{i}}$ | $\boldsymbol{ME(}{\hat{\boldsymbol{P}}}_{\boldsymbol{i}}\boldsymbol{)}$ | $\boldsymbol{CV(}{\hat{\boldsymbol{P}}}_{\boldsymbol{i}}\boldsymbol{)}$ | ${\hat{\boldsymbol{Y}}}_{\boldsymbol{i}}$ |
| --- | --- | --- | --- | --- | --- | --- | --- |
| 1 | Gujarat | 0.128 | 0.008 | (0.113,0.143) | 0.015 | 5.87 | 2582070 |
| 2 | Haryana | 0.044 | 0.003 | (0.038,0.050) | 0.006 | 7.43 | 277183 |
| 3 | Jammu & Kashmir | 0.161 | 0.011 | (0.139,0.183) | 0.022 | 6.95 | 520400 |
| 4 | Madhya Pradesh | 0.135 | 0.006 | (0.123,0.147) | 0.012 | 4.53 | 3930275 |
| 5 | Maharashtra | 0.213 | 0.004 | (0.205,0.222) | 0.008 | 1.98 | 4178481 |
| 6 | Mizoram | 0.211 | 0.011 | (0.189,0.233) | 0.022 | 5.38 | 10093 |
| 7 | Odisha | 0.297 | 0.010 | (0.277,0.316) | 0.019 | 3.33 | 3073889 |
| 8 | Punjab | 0.104 | 0.005 | (0.095,0.114) | 0.009 | 4.48 | 683487 |
| 9 | Telangana | 0.006 | 0.004 | (0.002,0.014) | 0.008 | 70.60 | 50652 |
| 10 | West Bengal | 0.361 | 0.021 | (0.321,0.402) | 0.041 | 5.73 | 7117080 |

$\hat{P}_{i}$: Estimated value of DIVA positivity rate; $SE(\hat{P}_{i})$: Estimated value of standard error of $\hat{P}_{i}$; 95% CI of $\hat{P}_{i}$: 95% confidence interval of $\hat{P}_{i}$; $ME(\hat{P}_{i})$: Estimated value of error margin for $\hat{P}_{i}$; $CV(\hat{P}_{i})$: co-efficient of variation of $\hat{P}_{i}$; $\hat{Y}_{i}$: estimated number of DIVA positive animals in each state

**Table S14**. Estimated values of sero-prevalence rates for FMD surveillance in India.

| **Year** | **Seroprevalence rate (%)** | **SE** | **95% CI (%)** | **CV (%)** | **ME (%)** | **Predicted Total** |
| --- | --- | --- | --- | --- | --- | --- |
| 2008 | 31.40 | 0.037 | (24.19, 38.66) | 11.74 | 7.23 | 4.55E+07 |
| 2009 | 33.20 | 0.037 | (25.97, 40.44) | 11.12 | 7.23 | 4.55E+07 |
| 2010 | 34.40 | 0.026 | (29.40, 39.44) | 9.37 | 5.02 | 4.68E+07 |
| 2011 | 30.80 | 0.029 | (25.14, 36.45) | 8.91 | 5.66 | 4.17E+07 |
| 2012 | 31.27 | 0.028 | (25.81, 36.73) | 10.87 | 5.46 | 4.21E+07 |
| 2013 | 42.69 | 0.046 | (33.60, 51.79) | 10.68 | 9.10 | 5.72E+07 |
| 2014 | 33.92 | 0.036 | (26.82, 41.01) | 13.56 | 7.10 | 4.55E+07 |
| 2015 | 29.61 | 0.040 | (21.71, 37.51) | 10.91 | 7.87 | 3.94E+07 |
| 2016 | 29.58 | 0.032 | (23.30, 35.90) | 14.97 | 6.33 | 3.92E+07 |
| 2017 | 26.28 | 0.039 | (18.57, 34.00) | 17.80 | 7.71 | 3.47E+07 |
| 2018 | 25.36 | 0.045 | (16.51, 34.21) | 22.10 | 8.85 | 3.43E+07 |
| 2019 | 27.10 | 0.060 | (15.39, 38.91) | 22.10 | 11.76 | 3.55E+07 |
| 2020 | 21.75 | 0.059 | (10.24, 33.25) | 26.99 | 11.51 | 2.93E+07 |
| 2021 | 18.27 | 0.022 | (14.02, 22.52) | 11.87 | 4.25 | 2.46E+07 |

$SE$: Estimated value of standard error; 95% CI: 95% confidence interval; $M$: Estimated value of error margin; $CV$: co-efficient of variation; Predicted total: predicted total of DIVA positive animals (having a history of infection)

**Supplementary Document S2:** User manual of ‘FMDSeroSurv’ R software package

**Description**:

**Package**: FMDSeroSurv

**Type**: Package

**Title**: Estimation of DIVA positive Rates for FMD Virus Sero-Surveillance

**Version**: 0.1.0

**Author**: Samarendra Das

**Maintainer**: Samarendra Das <samarendra.das@icar.gov.in>

**Description**: Provides state and national level estimates of Differentiating Infected from Vaccinated Animals (DIVA) along with other measures. Also, predicts the state and national level DIVA positive animal numbers.

**License**: GPL-3

**Encoding**: UTF-8

**Lazy Data**: true

**Depends**: R (>= 4.0.0)

**Imports**: stats

**RoxygenNote**: 7.2.1

**Built**: R 4.2.1; 2022-08-29 05:33:44 UTC; windows

**Availability:** https://github.com/sam-dfmd/ FMDSeroSurv

**Dependent packages:** stats, MASS

**Installation on Desktop/Laptop PC**:

Install the ‘FMDSeroSurv’ R package in R environment installed on your Desktop/Laptop PC using the following command.

library(devtools)

install_github("sam-dfmd/FMDSeroSurv")

Check whether the FMDSeroSurv R package is properly installed or not using the command.

library(FMDSeroSurv)

***Estimation of Sero-Surveillance parameters***

**Function**:

**>** FMDSeroSurv

**Description:** This function estimates and returns state and national level parameters and the related measures for sero-surveillance.

**Usage:** FMDSeroSurv(SeroSurvData, Census_Data)

**Arguments**

| **Inputs** | **Descriptions** |
| --- | --- |
| SeroSurvData | NSP-Ab antibody n X 3 data frame (n: number of states in the sample) obtained from sero-surveillance, where row represents the states, first column represents the total sample collected from each state and third column represents the number of DIVA positive samples. |
| Census_Data | Animal census N X 1 data frame (state wise bovine population), where rows are states and column is the bovine population (cattle + buffalo) (e.g. N = 28 before 2014 & N= 29 after 2014) |

**Output**

| **Sl. No.** | **Descriptions** |
| --- | --- |
| 1 | Returns DIVA_Positive, Var_DIVA_Prop., StError_Prop., MarginErr_Prop., CI_Lower, CI_Upper, Pred_Total are the DIVA positive rates (proportion as estimator), variance of the estimator, standard error, margin of error, 95 percentage CI and predicted total of animals having history FMD infection at the individual state-level. |
| 2 | Returns DIVA_Pos_Prop, Pos Rate, StError, CV, MarError, Lower_CI, Upper_CI, PredNatTotal, SE_PredTotal are the DIVA positive proportion, rates, its standard error, margin of error, co-efficient of variation, 95 percentage CI, predicted total, standard error of predicted total at the country/national level. |

**Example**

set.seed(23)

SeroSurvData = data.frame(round(runif(5, 100, 200)), rnbinom(5, mu = 40, size = 1), row.names = c("AP", "KN", "MH", "OD", "Guj")) colnames(SeroSurvData) <- c("Samples", "Positive")

(NB: SeroSurvData is a random sero-surveillance data generated for illustration purpose)

Census_Data <- data.frame(round(runif(10, 400, 600)), row.names=c("AP", "KN", "MH", "OD", "Guj", "Goa", "Raj", "Assam", "Miz", "Meg"))

colnames(Census_Data) <- "Total"

(NB: Census_Data is a random census data generated for illustration purpose only)

result <- FMDSeroSurv (SeroSurvData, Census_Data)

state_estimate <- result$StateEstimates

> state_estimate

DIVA_Positive(%) Var_DIVA_Prop. StError_Prop. MarginErr_Prop.(%) 95%_CI_Low 95%_CI_Upper Pred_Total

AP 11.39 0.000460 0.021 4.20 0.07 0.16 63

KN 50.00 0.001590 0.040 7.80 0.42 0.58 262

MH 2.26 0.000118 0.011 2.13 0.00 0.04 10

OD 15.79 0.000491 0.022 4.34 0.11 0.20 73

Guj 8.79 0.000271 0.016 3.23 0.06 0.12 41

Nat_estimate <- result$NationalEstimates

> Nat_estimate

DIVA_Pos_Prop Pos Rate(%) StError CV(%) MarError(%) Low_95%_CI Upper_95%_CI PredNatTotal SE_PredTotal

0.176 17.550 0.061 34.950 12.020 0.060 0.300 898.000 313.880

|  |  |  |  |  |  |  |  |  |  |  |
| --- | --- | --- | --- | --- | --- | --- | --- | --- | --- | --- |
|  | | | | | | | | | | |
|  | | | | | | | |  |  |  |

**Supplementary Figures:**


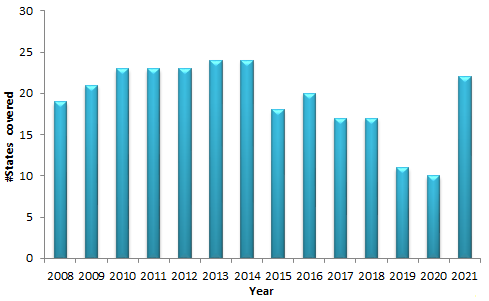


**Figure S1**: Number of states considered in Year-wise (2008-21) FMDV Sero-surveillance in India.

Distribution of the number of states covered under FMDV sero-surveillance activities during the year 2008-2021 is shown. In this bar graph, the X-axis represents the year from 2008-21 and Y-axis represents the number of states covered in the respective year. The following graph shows a familiar trend up to 2018 but in case of year 2019 and 2020 the number of states sharply declines due to the COVID-19 pandemic. However, in 2021 the trends bounce back to usual as sufficiently large number of states are considered in FMDV sero-surveillance activities.


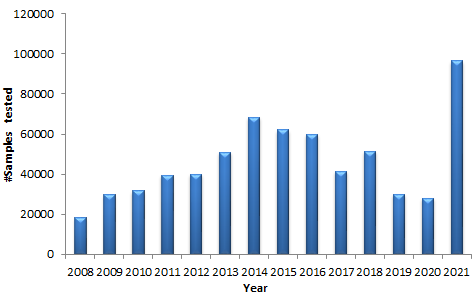


**Figure S2:** Number of serum samples considered in Year-wise (2008-21) FMDV Sero-surveillance activities in India.

The distribution of the serum samples tested at the ICAR-DFMD campuses under FMD sero-surveillance from year 2008-2021 is shown above. In this bar graph the X-axis represents the year from 2008-2021 and Y-axis represents the number of serum samples tested in respective year. The following graph shows a gradual increase up to 2014 then it slightly declines but in case of year 2019 and 2020 the number of tested samples sharply declines due to COVID-19 pandemic and corresponding nationwide lockdown. In 2021, FMD sero-surveillance came back to more rigorous and better form as highest number of samples are tested and also large number of states are considered in this year for surveillance.


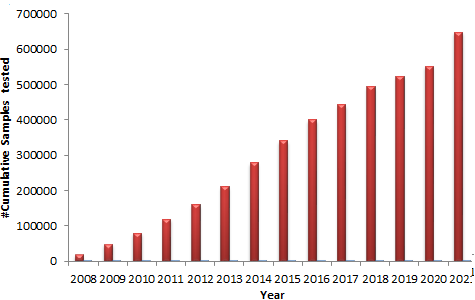


**Figure S3:** Cumulative number of serum samples considered in Year-wise (2008-21) FMDV Sero-surveillance activities in India.

The distribution of the cumulative serum samples, tested at ICAR-DFMD campuses under the FMDV sero-surveillance activities in India during the year 2008-21, is shown. In this bar graph the X-axis represents the year from 2008-2021 and Y-axis represents the cumulative serum samples tested in respective year. The following graph shows a gradual increase in samples testing up to 2020. But in 2021, there is a sharp increase in serum sample testing, indicating the improvement in diagnostic program for FMD sero-surveillance. In other words, so far ~0.7 million serum samples tested at ICAR-DFMD campuses for FMDV sero-surveillance in India (Figure S3). This milestone was achieved by ICAR-DFMD through its collaborative centres for FMD research, which are located almost in every states.


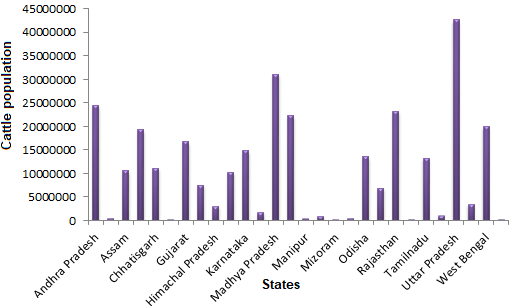


**Figure S4:** Bovine population in Indian States for the year 2007 census.

The distribution of the cattle population in major Indian states is shown in the above diagram. In this bar graph the X-axis represents the states and Y-axis represents the total cattle population in respective states. The following graph shows the population size comprising of cattle and buffalo, the above mentioned data are taken from the census data (2007) provided by the DAHD, Ministry of agriculture. The distribution shows the states including UP, MP, AP, and WB has higher bovine population compared to other states due to their large size and large farming population.


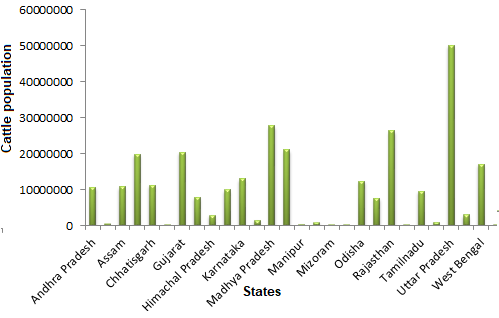


**Figure S5:** Bovine population in Indian States for the year 2012 census.

The distribution of the cattle population in major Indian states is shown in the above diagram. In this bar graph the X-axis represents the states and Y-axis represents the total cattle population in respective states. The following graph shows the population size comprising of cattle and buffalo, the above mentioned data are taken from the census data (2012) provided by the DAHD, Ministry of agriculture. The distribution shows the states including UP, MP, AP, and WB has higher bovine population compared to other states due to their large size and farming population.


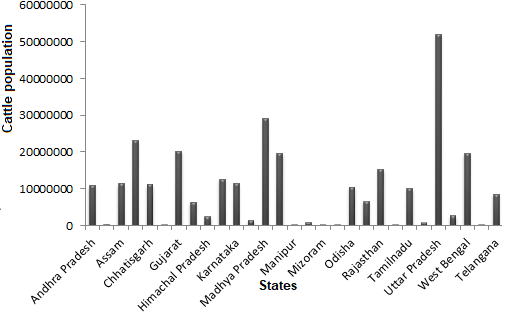


**Figure S6:** Bovine population in Indian States for the year 2019 census.

The distribution of the cattle population in major Indian states is shown in the above diagram. In this bar graph the X-axis represents the states and Y-axis represents the total cattle population in respective states. The following graph shows the population size comprising of cattle and buffalo, the above mentioned data are taken from the census data (2019) provided by the DAHD, Ministry of agriculture and farmers welfare. The distribution shows the states including UP, MP, AP, and WB has higher bovine population compared to other states due to their large size and large farming population.


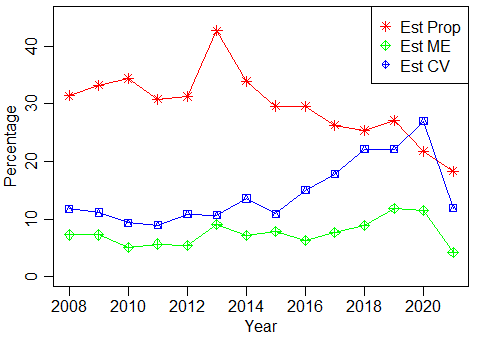


**Figure S7:** Year-wise distribution of estimated values of India’s DIVA positive rates.

The distribution of national level estimates of DIVA positive rates, margin of error, and Co-efficient variation (CV) values is shown for the years 2008-21. Here, the red color line graph represents the estimated DIVA positive rates, Blue color graph represents the estimated margin of error. Green color graph represents the estimated coefficient of variation values. In this line graph the X-axis represents the year and Y-axis represents the percentage of the measures in respective years. The following graph shows a sharp increase in DIVA positivity in year 2013 and gradually declining towards 2021.There is an increase trend in CV values over the years, but sharp increase was observed during 2020, due to COVID19 pandemic and nation-wide lock down. Further, in 2021, a lower value of DIVA rate with lower CV and margin of error was observed showing a lesser FMD infection in India with strong and robust system of FMDV sero-surveillance.


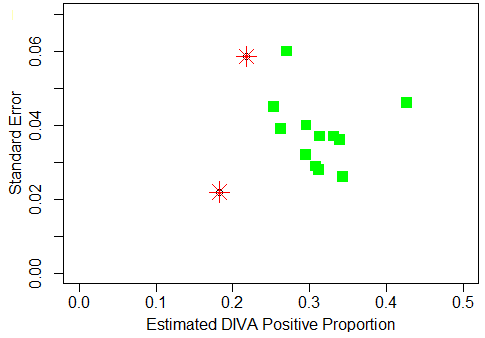


**Figure S8:** Scatter plot for estimated DIVA positive proportion and their standard error.

The plot shows the relationship between the estimated DIVA positive proportion and their standard error during pre and post COVID era. Here, the red and green colors show the distribution in post and pre COVID19 era respectively. In this graph, X-axis represents estimated DIVA positive proportion and Y-axis represents the standard error. During the COVID period like 2020, the DIVA positive rate is lesser due to restricted animal movement in the nation-wide lock down. However, the standard error for this year is high due to lesser samples and states are included in the FMDV sero-surveillance. Further, in 2021, the DIVA positive proportion is least compared to previous years with lowest standard error. This indicates reduced natural infection in bovine population and a robust and efficient sero-surveillance system is in place.


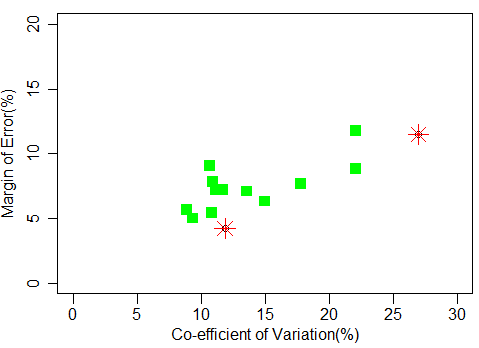


**Figure S9:** Scatter plot for CV and margin of error of the estimated DIVA positive proportions.

The plot shows the relationship between the CV and margin of error of the estimated DIVA positive proportions during pre and post COVID era. In this graph, X-axis represents estimated CV (%) of the DIVA positive proportions and Y-axis represents the estimated margin of error in the DIVA positive proportions. Here, the red and green colors show the distribution in post and pre COVID19 era respectively. The relation between margin of error and CV is linear. During the COVID period like 2020, the margin of error and CV is high due to restricted FMDV sero-surveillance due to the nation-wide lock down. Also, this indicates the field staffs could not collect not much serum samples due to restricted movements. In 2021, normalcy has arrived and a strict and robust FMDV sero-surveillance was undertaken, which reflected in the lower CV and margin of error.


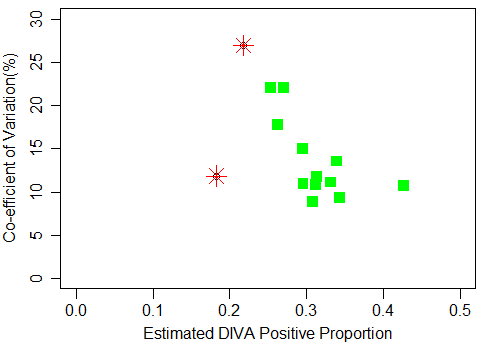


**Figure S10:** Scatter plot for estimated DIVA positive proportions and their CV.

The plot shows the relationship between the estimated DIVA positive proportions and their CV values during pre and post COVID era. In this graph, X-axis represents estimated DIVA positive proportions and Y-axis represents the estimated CV in %. Here, the red and green colors show the distribution in post and pre COVID19 era respectively. During the COVID period like 2020, the DIVA positive rate is lesser due to restricted animal movement in the nation-wide lock down. However, the CV for this year is highest due to lesser samples and states are included in the FMDV sero-surveillance. Further, in 2021, the DIVA positive proportion is least compared to previous years with lower CV. This indicates reduced natural infection in bovine population and a robust and efficient sero-surveillance system is in place.
